# The genus *Scrophularia:* a source of iridoids and terpenoids with a diverse biological activity

**DOI:** 10.1080/13880209.2017.1397178

**Published:** 2017-11-10

**Authors:** Ardalan Pasdaran, Azadeh Hamedi

**Affiliations:** a Medicinal Plants Processing Research Center, Shiraz University of Medical Sciences, Shiraz, Iran;; b Department of Pharmacognosy, School of Pharmacy, Shiraz University of Medical Sciences, Shiraz, Iran

**Keywords:** Scrophulariaceae, phytochemistry, folk medicine, ethnopharmacology, biological activity, natural product, hepatoprotective, antimicrobial, phenylpropanoid, Phenylethanoid

## Abstract

**Context:** *Scrophularia* genus (Scrophulariaceae) includes about 350 species commonly known as figwort. Many species of this genus grow wild in nature and have not been cultivated yet. However, some species are in danger of extinction.

**Objective:** This paper reviews the chemical compounds, biological activities and the ethnopharmacology of some *Scrophularia* species.

**Materials and methods:** All information was obtained through reported data on bibliographic database such as *Scopus*, United States National Agricultural Library, Biological Abstracts, EMBASE, PubMed, MedlinePlus, PubChem and Springer Link (1934–2017). The information in different Pharmacopoeias on this genus was also gathered from 1957 to 2007.

**Results*:*** The structures of 204 compounds and their biological activity were presented in the manuscript: glycoside esters, iridoid glycosides and triterpenoids are the most common compounds in this genus. Among them, scropolioside like iridoids have shown potential for anti-inflammatory, hepatoprotective and wound healing activity. Among the less frequently isolated compounds, resin glycosides such as crypthophilic acids have shown potent antiprotozoal and antimicrobial activities.

**Conclusion:** The *Scrophularia* genus seems to be a rich source of iridoids and terpenoids, but isolation and identification of its alkaloids have been a neglected area of scientific study. The diverse chemical compounds and biological activities of this genus will motivate further investigation on *Scrophularia* genus as a source of new therapeutic medications.

## Introduction

The Scrophulariaceae family consists of 220 genera. *Scrophularia* genus is one of the large genera of the Scrophulariaceae. Distribution of these genera occurs mainly through mountainous regions (e.g., *Scrophularia farinosa* Boiss. and *Scrophularia amplexicaulis* Benth.) to rarely in deserts (e.g., *Scrophularia deserti* Delile). This genus is represented by 60 species in the flora of Iran and can be used as heart stimulant, circulatory stimulant and diuretic. Other traditional uses of this genus include antipyretic, febrifuge, antibacterial, anti-erythema, anticonstipation, antifurunculosis, ulcerous stomatitis and tonsillitis treatment.

**Figure 1.**
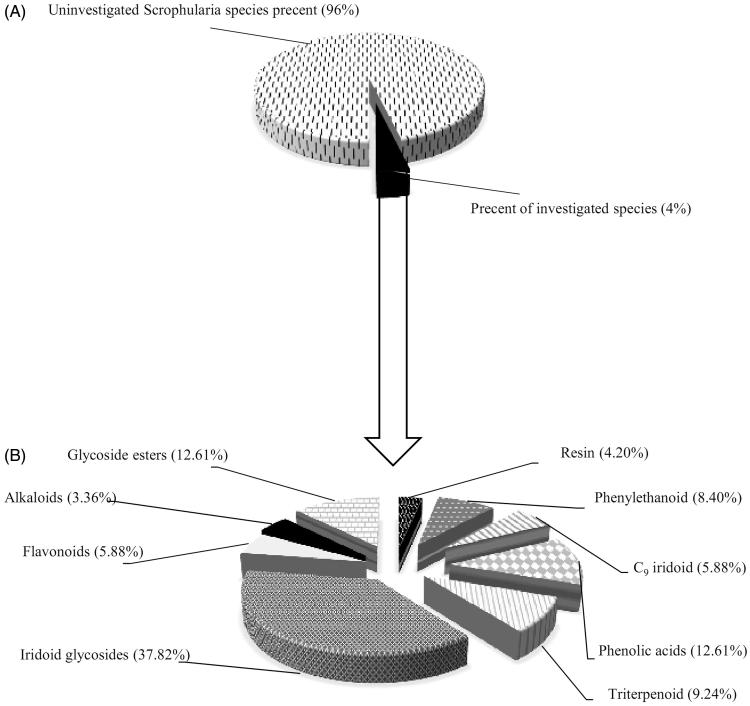
Comparison between chemical compounds isolated from investigated *Scrophularia* species. Part (A) shows investigated species percentage against the total species. Part (B) shows the relative percent of the various phytochemical class isolated from investigated *Scrophularia* species.

Among these traditional uses of the *Scrophularia*, anti-inflammatory and anti-infections’ treatment in different types of diseases is common (Viola 1966; Swiatek and Dombrowicz 1975). The therapeutic potential of the *Scrophularia* has led researchers to focus on the isolation and determination of their bio-active compounds. Some of these species are characterized mainly by glycoside esters or phenylpropanoid glycosides (Calis et al. 1988b; de Santos et al. 2000; Li et al. 2000, 2009), saponins, and iridoids (Çalis et al. 1993a; Yamamoto et al. 1993; Pachaly et al. 1994; Maksudov et al. 1996; Bhandari et al. 1997; Chen et al. 2007; Chebaki et al. 2011). According to some findings, phenylpropanoid glycosides and iridoids are the major part of *Scrophularia* genus secondary metabolites, which showed apparent therapeutic potential in numerous investigations (Figure 1). Several biological effects of phenylpropanoid such as antioxidants, hepatoprotective, antitumor, anti-inflammatory and other useful effects have been studied over the past few years (Garrido et al. 2004; Korkina et al. 2007). Another main class of secondary metabolites is iridoids compounds which constitute the most chemical and biological diversity in *Scrophularia* genus. The several reported biological activities of these compounds have led to increased inclination for the isolation of these classes of chemical compounds (Garg et al. 1994; Giner et al. 2000; Kim and Kim 2000; Kim et al. 2002a; Lee et al. 2002; Stevenson et al. 2002; Kim et al. 2003a; Tasdemir et al. 2005; Valiyari et al. 2012). Based on data extracted from different studies, most biological activities of iridoids include anti-inflammatory, anticancer and antiprotozoal (Dinda et al. 2009). This review presents a brief case for the medicinal uses and the phytochemical and pharmacological properties of the *Scrophularia* genus.

## Materials and methods

All information regarding the chemical and biological activity of the plants were obtained through reported data from 1934 to 2017 on bibliographic database such as Scopus, United States National Agricultural Library, Biological Abstracts, EMBASE, PubMed, MedlinePlus, PubChem and Springer Link. The search keywords without any language limitation were *Scrophularia*, biological activity, traditional uses, iridoids, phenylethanoids, alkaloids, resin glycosides, triterpenoid glycoside, essential oils and diterpenoids. The gathered information was then compared with data reported in recent publications (the last 17 years, 2000–2017), and the Pharmacopoeia of the People's Republic of China. Also, data collection on different Pharmacopeias including British Herbal Pharmacopoeia, the Japanese Pharmacopoeia, the French Pharmacopoeia and the Pharmacopoeia of the Royal College of Physicians at Edinburgh (1957–2007) was carried out in order to create a pharmaceutical overview about these species.

## Results

### Biology and ethnopharmacology

Most *Scrophularia* species are annual or perennial herbaceous plants, with woody base and rarely suffruticose, and can also be spinose in rare cases. However, a few of this genus are sub-shrubs. Flowers are urceolate or tubulose. The length of corolla ranges from 3 to 20 mm. Lips are equal or unequal, which is one of the important characteristics for distinguishing species. With thyrse inflorescence or in rare cases, racemose with one or two flower in each cyme, mostly have four-angled stems and opposite leaves. Some *Scrophularia* species are widely used as traditional medicine. Several countries, including China, Korea and Japan, have used these species as traditional therapeutics as anti-inflammatory and anticancer remedies. Roots of *S. ningpoensis* Hemsl. “Xuan Shen”, *S. buergeriana* Miquel, Ann. Mus. Bot. Lugduno-Batavi. “Beixuan Shen” and *S. nodosa* L. (common figwort) have been used as therapeutic remedies in fever, swelling, constipation, pharyngitis, neuritis and laryngitis. In Europe, other species, such as *S. aquatica* L. (water figwort), are used as laxatives, heart stimulants, circulatory stimulants and diuretics. In ancient Iranian medicine, roots and aerial parts of *S. lucida* L. “Sinderitis” and *S. chryasanthemifolia* Bory & Chaub. “Heterasinderitis” are used as heart and circulatory stimulants. Table 1 shows *Scrophularia* species which are used traditionally as therapeutic remedy.

**Table 1.** The traditional use of *Scrophularia* species mentioned in different pharmacopoeias.

Name	Plant medicinal part	Traditional uses	Pharmacopeia	Other references
*S. ningopoensis* “Xuan Shen” (Chinese figwort)	Roots	Anti-inflammatory, treatment of cancer and antioxidant	Pharmacopoeia of the People’s Republic of China (Commission 2005) Society of Japanese Pharmacopoeia (Pharmacopoeia 2006)	Marty (1999), Wang et al. (2005) and Zhu (1998)
*S. aquatica* (water figwort) * S. marilandica* (late figwort)	Roots and aerial parts	Laxative, heart stimulant, circulatory stimulant and diuretic	The Pharmacopoeia of the Royal College of the Physicians at Edinburgh, Materia Medica (Lewis et al. 1748) French Pharmacopoeia (Ministry of Health 2012)	Marty (1999)
*S. buergeriana* “Bei xuan shen”	Roots	Treatment of fever, swelling, constipation, pharyngitis, neuritis and laryngitis	Pharmacopoeia of the People’s Republic of China (Commission 2005)	Pinkas et al. (1994) and Wang et al. (2005)
*S. dentata* “Ye-Xin-Ba” (Tabatian figwort)	Aerial parts	Treatment of smallpox, measles, high-heat plague and poisoning	Pharmacopoeia of the People’s Republic of China (Commission 2005)	Zhang et al. (2014)
*S. nodosa* (common figwort)	Roots and aerial parts	Treatment of Fever, swelling, constipation, pharyngitis, neuritis and laryngitis	French Pharmacopoeia (Ministry of Health, 2012) British Herbal Pharmacopoeia (Willoughby et al. 1996)	Zhu (1998)
*S. lucida* L. ”Sinderitis”	Roots and aerial parts	Heart stimulant, circulatory stimulant, diuretic	–	Goodyer and Gunther (1968)
*S. chryasanthemifolia* L. (Hetera sinderitis)	Roots and aerial parts	Heart stimulant, circulatory stimulant and diuretic	–	Goodyer and Gunther (1968)
*S. canina* “a ruta salvacce” (Ruta canina)	Roots	Treatment of dermatitis and rheumatoid arthritis	–	Berdini et al. (1991), Guarrera and Lucia (2007) and Pieroni et al. (2004)

### Phytochemistry

From the genus *Scrophularia*, chemical compounds such as flavonoids, phenylethanoids and glycoside esters, phenolic acids, C_9_ iridoid, glycosides, resin glycosides and fatty acids derivatives, triterpenes, triterpenoid glycosides, alkaloids, diterpenoids and essential oils can be isolated (Tables 2 and 3 and Figure 1). As mentioned above, some of these chemical substances produce bioactivities in various models (Table 4).

**Table 2.** Compounds isolated from the genus Scrophularia (the structure of the compounds illustrated in text).

Plant name	Compound	No.	Ref.
*S. auriculata*	Scrovalentinoside	**130**	(Giner, et al. 2000, Giner et al. 1998)
	Verbascosaponin A	**188**	
	Scropolioside A	**134**	
	Ilwensisaponin A	**177**	
	Verbascoside	**48**	
*S.amplexicaulis*	Scropolioside D	**131**	(Pasdaran, et al. 2016)
	Scrophuloside B_4_	**117**	
	Salidroside	**75**	
	Verbascoside	**48**	
	Eugenol	**200**	
	Eugenol acetate	**203**	
	1-Octen-3-ol	**204**	
*S. buergeriana*	Buergerinin F	**78**	(Kim and Kim 2000, Lin, et al. 2000, Kim, et al. 2002b, Kim, et al. 2003b, Jeong et al. 2008, Yan and Xie 2011)
	Buergerinin G	**79**	
	Buergerinin E	**80**	
	Ningpogenin	**86**	
	Buergerinin D	**82**	
	Buergerinin C	**84**	
	Buergerinin B	**85**	
	8-*O*-E-p-methoxycinnamoyl harpagide	**102**	
	8-*O*-Z-p-methoxycinnamoyl harpagide	**103**	
	6′-*O*-E-p-methoxycinnamoyl harpagide	**104**	
	6′-*O*-Z-p-methoxycinnamoyl harpagide	**105**	
	*Trans*-cinnamic acid	**11**	
	*(E)*-*p*-methoxycinnamic acid	**12**	
	*(E)*-*p*-methoxycinnamic acid methyl ester	**40**	
	*(E)*-o-methoxycinnamic acid	**10**	
	*(E)*-*p*-coumaric acid	**16**	
	*(E)*-caffeic acid	**41**	
	*(E)*-ferulic acid	**42**	
	Homovanilline alcohol	**36**	
	Buergeriside A_1_	**67**	
	Buergeriside B_1_	**66**	
	Buergeriside B_2_	**65**	
	Buergeriside C_1_	**64**	
	Harpagoside	**113**	
*S. canina*	7,8-Didehydro-6b,10-dihydroxy-11-noriridomyrmecin	**83**	(Berdini, et al. 1991, Venditti et al. 2015)
	8-*epi*-Loganic acid	**138**	
	Verbascoside	**48**	
	(*E*)-Phytol	**174**	
*S. cryptophila*	Crypthophilic acid A	**171**	(Tasdemir, et al. 2008)
	Crypthophilic acid B	**172**	
	Crypthophilic acid C	**173**	
	Buddlejasaponin III	**182**	
	8-*O*-Acetyl harpagide	**100**	
	Harpagide	**114**	
*S. dentata*	Scrodentoside A	**139**	(Zhang, et al. 2015b, Zhang, et al. 2014)
	Scrodentoside B	**140**	
	Scrodentoside C	**141**	
	Scrodentoside D	**142**	
	Scrodentoside E	**143**	
	Scrodentoside F	**154**	
	Scrodentoside G	**155**	
	Scrodentoside H	**156**	
	Scropolioside G	**157**	
	Scropolioside H	**158**	
	Saccatoside	**159**	
	6-*O*-Methyl catalpol	**94**	
	Catalpol	**93**	
	6′-*O*-E-p-feruloyl harpagide	**107**	
	Scropolioside D	**131**	
	*Cis*-harpagoside	**144**	
	Harpagoside	**113**	
	Laterioside	**101**	
	Scorodioside	**145**	
	6-*O*-α-*L*-(4″-*O*-trans-cinnamoyl)-rhamnopyranosylcatalpol	**146**	
	6-*O*-α-*L*-(4″-*O*-trans-p-coumaroyl)-rhamnopyranosylcatalpol (Scropolioside F)	**147**	
	lagotisoside D	**148**	
	8-*O*-Acetyl harpagide	**100**	
	7-Deoxygardoside	**149**	
	Ajugoside	**89**	
	8-*epi*-deoxyloganic acid	**150**	
	6′-*O*-p-Coumaroyl harpagide	**151**	
*S. dentata* (continued)	10-Deoxygeniposidic acid	**152**	
	Geniposidic acid	**153**	
	Ajugol	**90**	
	Harpagide	**114**	
	Scrodentoid A	**195**	
	Scrodentoid B	**196**	
	Scrodentoid C	**197**	
	Scrodentoid D	**198**	
	Scrodentoid E	**199**	
	Lipedosides A-I	**51**	
	Osmanthuside B	**52**	
	Martynoside	**53**	
	Diacetylmartynoside	**54**	
	Verbascoside	**48**	
	Isoverbascoside	**49**	
	3-*O*-*trans*-Feruloylrhamnopyranose	**76**	
	2-*O*-*trans*-Feruloylrhamnopyranose	**77**	
*S. deserti*	3-(*R*)-1-Octan-3-yl-3-*O*-β-D-glucopyranoside	**169**	(Ahmed, et al. 2003, Stavri, et al. 2006)
	3(ζ)-Hydroxy-octadeca-4(*E*), 6(*Z*)-dienoic acid	**170**	
	6-*O*-α-L-rhamnopyranosylcatalpol	**97**	
	Buddlejoside A_8_	**98**	
	Harpagoside B	**99**	
	8-*O*-Acetyl harpagide	**100**	
	Koelzioside	**132**	
	Scropolioside D	**131**	
	Scropolioside D_2_	**133**	
	Scropolioside B	**135**	
	Scrospioside A	**136**	
	Laterioside	**101**	
*S. frutescens*	(Z)-*p*-Coumaric acid	**13**	(Fernandez, et al. 1998, Garcia, et al. 1998)
	(Z)-Caffeic acid	**14**	
	(Z)-Isoferulic acid	**15**	
	(Z)-p-Methoxycinnamic acid	**16**	
	(E)-p-coumaric acid	**17**	
	(E) 3, 4-Dimethoxy cinnamic acid	**18**	
	(Z) Ferulic acid	**19**	
	(Z)-Methoxycinnamic acid methyl ester	**20**	
	Syringic acid	**21**	
	Gentisic acid	**22**	
	Protocatechuic acid	**23**	
	Isovanillic acid	**24**	
	Catalpinic acid	**25**	
	Vanillic acid	**26**	
*S. ilwensis*	Ilwensisaponin A (Mimengoside A)	**177**	(Çalis, et al. 1993a, Çalis, et al. 1993b)
	Ilwensisaponin B	**178**	
	Ilwensisaponin C	**179**	
	Ilwensisaponin D	**180**	
	Karsoside	**116**	
	Scropolioside D	**131**	
	Aucubin	**109**	
	Harpagide	**114**	
	8-*O*-Acetylharpagide	**100**	
	Ajugol	**90**	
	Angoroside C	**46**	
	Quercetin-3-*O*-rutinoside	**7**	
	Kaempferol-3-*O*-rutinoside	**8**	
*S. kakudensis*	Songarosaponin A	**189**	(Yamamoto A 1993)
	Saksisaponin A	**181**	
	Buddlejasaponin I	**182**	
	Buddlejasaponin II	**183**	
	Buddlejasaponin III	**184**	
	Scrophulasaponin II	**185**	
	Scrophulasaponin III	**186**	
	Scrophulasaponin IV	**187**	
*S. koelzii*	Koelzioside	**132**	(Bhandri et al. 1992, Garg, et al. 1994, Bhandari, et al. 1996, Bhandari, et al. 1997)
	Scropolioside A	**134**	
	Scropolioside B	**135**	
	6-*O*-(3”-*O*-*p*-Methoxy-cinnamoyl)-α-L-rhmanopyranosylcatalpol	**161**	
	Scrokoelziside A	**175**	
	Scrokoelziside B	**176**	
*S. lepidota*	Ajugoside	**89**	(Tasdemir, et al. 2005)
	Ajugol	**90**	
	Sinuatol	**91**	
	6-*O*-β-D-Xylopyranosylaucubin	**92**	
*S. lepidota* (continued)	Catalpol	**93**	
	6-*O*-Methyl catalpol	**94**	
	3,4-Dihydro-methyl catalpol	**95**	
	1-Dehydroxy-3,4-dihydro aucubigenin	**96**	
	Scrolepidoside	**137**	
	Aucubin	**109**	
	Angoroside C	**46**	
	Ningpogenin	**86**	
*S. ningpoensis*	Haemoplantaginin	**4**	
	8-Hydroxycoumarin	**38**	
	6-Hydroxyindan-1-one	**39**	
	4-Methylcatechol	**35**	
	*trans*-Cinnamic acid	**10**	
	3-Methylphenyl-*O*-β-xylopyranosyl-(1→6)-*O*-β-glucopyranoside	**70**	(Kajimoto, et al. 1989, Qian, et al. 1991, Qian et al. 1992, Li, et al. 2000, Nguyen, et al. 2005, Chen, et al. 2007, Chen et al. 2008, Li, et al. 2009, Niu, et al. 2009, Zhang et al. 2012, Zhu et al. 2013, Zhang, et al. 2015a)
	4-Hydroxybenzaldehyde	**27**	
	3′-Hydroxyacetophenone	**28**	
	Scrokoelziside A	**175**	
	Buergeriside A1	**67**	
	Sibirioside A	**68**	
	Cistanoside F	**69**	
	Cistanoide D	**43**	
	6′-*O*-Caffeoyl harpagide	**106**	
	6′-*O*-*E-p*-Feruloyl harpagide	**107**	
	6″-*O*-β-Glucopyranosylharpagoside	**108**	
	8-*O*-Acetyl harpagide	**100**	
	β-Sitosterol	**192**	
	β-Sitosterol glucoside	**193**	
	Angoroside C	**46**	
	Nepitrin	**3**	
	Buergerinin A	**81**	
	Aucubin	**109**	
	Ningpogenin	**86**	
	Ningpogoside A	**87**	
	Ningpogoside B	**88**	
	4′-hydroxyacetophenone	**30**	
	3′,5′-Dimethoxy-4′-hydroxyacetophenone	**31**	
	3′-Methoxy-4′-hydroxyacetophenone	**32**	
	(*Z*)-4-Hydroxycinnamic acid methyl ester	**34**	
	(*E*)-p-Methoxycinnamic acid	**11**	
	*trans*-Caffeic acid methyl ester	**33**	
	Scropolioside B	**135**	
	Scrophularianine A	**164**	
	Scrophularianine B	**165**	
	Scrophularianine C	**166**	
	2,6-Dimethoxy-4-methoxymethylphenol	**37**	
	Homovanillic alcohol	**36**	
	Scrophuloside B_4_	**117**	
	Scrophuloside A_4_	**118**	
	6-*O*-Feruloylb-fructofuranosyl-(2→1)-*O*-α-glucopyranosyl-(6→1)-*O*-α-glucopyranoside	**74**	
	Scrokoelziside B	**176**	
	6-*O*-cinnamoyl b-fructofuranosyl-(2→1)-*O*-α-glucopyranosyl-(6→1)-*O*-α-glucopyranoside	**73**	
	Ningposide A	**61**	
	Ningposide B	**62**	
	Homoplantaginin	**9**	
	Eurostoside	**115**	
	2-(3-Hydroxy-4-methoxyphenyl)ethyl-*O*-α-arabinopyranosyl-(1→6)-*O*-α-rhamnopyranosyl-(1→3)-*O*-β-Glucopyranoside	**72**	
	Phenyl*O*-β-xylopyranosyl-(1→6)-*O*-β-glucopyranoside	**71**	
	Ningpoensines B/C	**163**	
	Vanillin	**29**	
	6-*O*-Methyl catalpol	**94**	
	8- *O*-Feruloylharpagide	**110**	
	8-*O*-(2-Hydroxycinnamoyl) harpagide	**111**	
	6-*O*-α-D-Galactopyranosylharpagoside	**112**	
	Harpagoside	**113**	
	Harpagide	**114**	
	Ningposide C	**60**	
	Ningposide D	**63**	
	Buergeriside C1	**64**	
	Buergeriside B2	**65**	
	Buergeriside B1	**66**	
	Ningpoensine A	**162**	
*S. oxysepala*	Scrokoelziside A	**175**	(Orangi et al. 2013, Orangi et al. 2016, Valiyari et al. 2012)
	Scrokoelziside B	**176**	
	Verbascosaponin	**177**	
	Harpagoside B	**99**	
	Scropolioside D	**131**	
	2-(4-chlorobenzyl amino) ethanol	**167**	
	Eugenol	**200**	
	Dehydroeugenol	**201**	
	Methyl benzyl alcohol	**202**	
	1-Octen-3-ol	**204**	
*S. nodosa*	Jionoside D	**50**	(Miyase and Mimatsu 1999, Stevenson et al. 2002, Swiatek 1972)
	Scrovalentinoside	**130**	
	Angoroside C	**46**	
	Scrophuloside A_2_	**120**	
	Scrophuloside A_4_	**118**	
	Scrophuloside A_5_	**121**	
	Scrophuloside A_6_	**122**	
	Scrophuloside A_7_	**123**	
	Scrophuloside A_8_	**124**	
	Scrophuloside A_1_	**119**	
	Buddlejoside A_5_	**126**	
	Buddlejoside A_3_	**127**	
	Buddlejoside A_4_	**129**	
	Pulverulentoside II	**125**	
	Scrophuloside A_3_	**160**	
	Verbascoside A	**128**	
	Scrophuloside B_1_	**57**	
	Scrophuloside B_2_	**58**	
	Purpureaside C	**56**	
	Verbascoside	**48**	
	Angoroside A	**44**	
	*cis*-Verbascoside	**59**	
*S. scopolii*	Angoroside A	**44**	(Calis et al. 1988a, Calis, et al. 1988b)
	Angoroside B	**45**	
	Angoroside C	**46**	
	Angoroside D	**47**	
	Verbascoside	**48**	
	Isoverbascoside	**49**	
	ScropoliosideA	**134**	
	ScropoliosideB	**135**	
*S. striata*	Quercetin	**1**	(Monsef-Esfahani, et al. 2010)
	*trans*-cinnamic acid	**11**	
	Isorhamnetin-3-*O*-rutinoside		
	Nepitrin	**3**	
	Verbascoside	**48**	
	1-Octen-3-ol	**204**	
*S. scorodonia*	8-*O*-Acetyl harpagide	**100**	(Emam et al. 1997, de Santos, et al. 2000, Bermejo, et al. 2002, Díaz, et al. 2004)
	Scrolepidoside	**137**	
	Saikosapoinin I (Buddlejasaponin IV)	**190**	
	Saikosapoinin II (Sandrosaponin I)	**191**	
	Isoangoroside C	**55**	
	Buddlejasaponin I	**182**	
*S. takesimensis*	Isorhamnetin-3-*O*-rutinoside	**2**	(Kim, et al. 2012a)
	Nepitrin	**3**	
	β-Sitosterol	**192**	
	α-Spinasterol 3-*O*-β-D-glucopyranoside	**194**	
	5-Hydroxypyrrolidin-2-one	**168**	
	*trans*-Cinnamic acid	**11**	
	*(E)-p*-Methoxycinnamic acid	**12**	
	*(E)-o*-Methoxycinnamic acid	**10**	
	Acacetin	**5**	
*S. trifoliata*	Catalpol	**93**	(Ramunno et al. 2006)
	Aucubin	**109**	

**Table 3.** Some of the *Scrophularia* species essential oil major compounds.

Species	Major compounds
*S. oxysepala*	Methyl benzaldehyde, methyl benzyl alcohol, 1-octen-3-ol, eugenol and phytol
*S. amplexcaulis*	Eugenol, 1-cten-3-ol, anethole, caryophyllene oxide and eugenol acetate
*S. striata*	1-octen-3-ol, banzyl banzoat, benzaldehyde, linalool and phytol
*S. frigida*	Oxygenated monoterpenes, L-linalool, geraniol, α-terpineol, and 1-octen-3-ol

**Table 4.** Pharmacological activities of some *Scrophularia* species.

Species	Biological activity	Responsible compound or extract	References
*S. amplexicaulis*	Antibacterial (aginest *S. aureus*)	Essential oil	Pasdaran et al. (2012, 2016)
	Antimalarial	Methanolic extract & fractions	
	Free radical scavengering activities and general toxicity	Methanolic extract & fractions	
*S. dentata*	Anti-inflammatory activity significantly inhibited CoA-induced splenocyte proliferation	Iridoids & Scrodentoids A–E, scropoliosides	Zhang et al. (2014, 2015b)
*S. auriculata*	Antibacterial	Phenolic acids	Cuéllar et al. (1998) and Giner et al. (2000)
	Anti-inflammatory	Iridoids and saponins, Hydroalcoholic extract	
*S. buergeriana*	Neuroprotective & Anti-amnestic	Chloroformic & methanolic extracts from roots, harpagoside and 8-*O*-E-p-methoxycinnamoylharpagide Phenylpropanoids & Phenolic acids	Kim and Kim (2000), Lee et al. (2002), Kim et al. (2003b), Jeong et al. (2008), and Kim et al. (2011, 2012b)
	Hepatoprotective		
	Anti-inflammatory		
*S. canina*	Insecticidal activity	Plant, phenolic acids	Germinara et al. (2011)
*S. cryptophila*	Antiprotozoal and antimycobacterial activities	Crypthophilic acid A, C & buddlejasaponin III, acetylharpagide	Tasdemir et al. (2008)
*S. deserti*	inhibiting an enzyme or enzymes of Type II fatty acid synthesis (FAS)	Unsaturated fatty acids, ethanolic extract	Ahmed et al. (2003), Stavri et al. (2006) and Bahmani et al. (2013)
	Anti-inflammatory	Scropolioside-D_2_ & harpagoside B	
	Antidiabetic	Scropolioside-D_2_ & harpagoside B	
*S. frutescens*	Antibacterial	Aerial part aqueous extract, phenolic acids	Fernandez et al. (1996, 1998) and Garcia et al. (1998)
	Anti-inflammatory	Phenolic acids, Iridoids	
	Cytostatic activity	Phenolic acids	
*S. grossheimi*	Hepatoprotective	1,6-di-*O*-caffeoyl-β-D-glucopyranose & flavonoids	Akhmadov et al. (1969), Akhmedov and Litvinenko (1969) and Galindez et al. (2001)
*S. koelzii*	Hepatoprotective & immunostimulant	Scropolioside-A, koelzioside, harpagoside, 6-*O*-(3″-*O*-p-Methoxy-cinnamoyl)-α-L-rhmanopyranosyl catalpol, chloroform fraction of the aerial parts	Garg et al. (1994)
*S. lepidota*	Anti-protozoal & Antiplasmodial	Ningpogenin, sinuatol	Tasdemir et al. (2005)
*S. ningpoensis*	Cardioprotective	Trans-caffeic acid methyl ester & 4-methylcatechol, 6″-*O*-caffeoylharpagide, 6″-*O*-(p-coumaroyl) harpagide, harpagoside and Phenylethanoide glycosides	Chen et al. (2008) and Zhu et al. (2013)
	Anti-inflammatory	Ningpogenin, ningpogoside A and ningpogoside B and hydrophilic extract	Qian et al. (1991)
	Antibacterial	Scrokoelziside A and ethanolic leave extract	Li et al. (2009)
*S. nodosa*	Wound healing activity	Scopolioside A, scrophuloside A4 and scrovalentinoside	Stevenson et al. (2002)
*S. oxysepala*	Insecticidal activity	Essential oil, methanolic fractions	Pasdaran et al. (2013, 2017) and
	Apoptosis	Dichloromethane and methanol extracts	Valiyari et al. (2012) and Orangi et al. (2013)
	Cytotoxic Free radical scavenging	Methanolic fractions, scropolioside D, harpagoside B &2-(4-chlorobenzyl amino) ethanol	Pasdaran et al. (2017)
*S. striata*	Wound healing and Anti-inflammatory	Ethanolic extract, ethyl acetate extract	Hajiaghaee et al. (2007) and Azadmehr et al. (2009)
	Antibacterial	Ethanolic extract	Benito et al. (1998) and Bahrami and Ali (2010)
	Antioxidant	Ethanolic extract	Díaz et al. (2004)
*S. scorodonia*	Anti-inflammatory	Angoroside A, angoroside C, angoroside D, acteoside, isoacteoside, Buddlejasaponin I& Saikosapoinin I, II	
	Antiviral	Scorodioside, Buddlejasaponin IV	Bermejo et al. (2002)
*S. takesimensis*	Strong aldose reductase (AR) inhibitory activity	Acacetin	Kim et al. (2012a)

### Flavonoids and flavonoid glycosides

**Figure 2.**
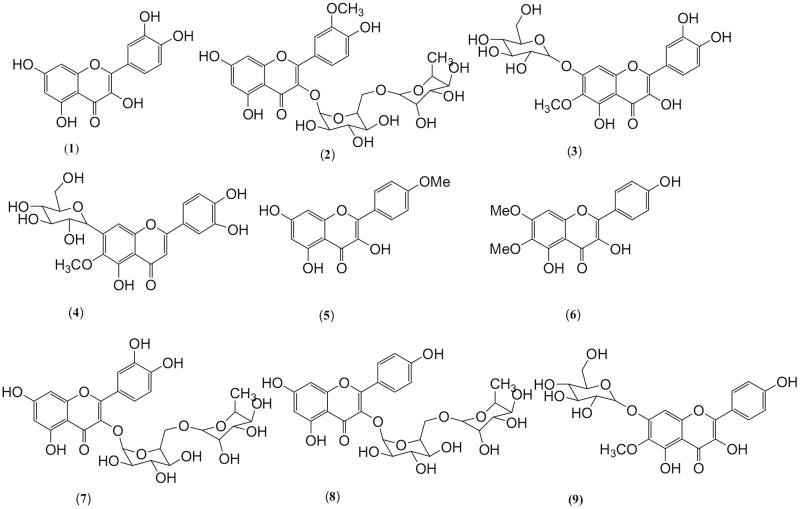
Isolated flavonoids and flavonoid glycosides from *Scrophularia* genus.

Although flavonoids are the major compounds in plants, and consist of the most dominant compounds in many of the plants family, *Scrophularia* genus is an exceptional case regarding the existence of flavonoids. Very negligible flavonoids compounds, such as quercetin (**1**), isorhamnetin-3-*O*-rutinoside (**2**), nepitrin (**3**) and haemoplantaginin (**4**) have been isolated from *S. striata* Boiss. *O*-methylated flavone and acacetin (**5**) have been isolated from endangered Korean species of *S. takesimensis* Nakai (Li et al. 2009; Monsef-Esfahani et al. 2010; Kim et al. 2012a). Other flavonoids such as scrophulein (**6**) and homoplantaginin (**9**) have been isolated from *S. grossheimii* Schischk. and *S. ningpoensis*, respectively (Akhmedov and Litvinenko 1969). An investigation on the bioactive compounds of *S. ilwensis* K.Koch. resulted in the isolation of quercetin-3-*O*-rutinoside (**7**) and kaempferol-3-*O*-rutinoside (**8**) from polar extract (Çalis et al. 1993a). Many bioactivities such as antioxidant, antibacterial, anti-inflammatory and antinociceptive activities have been reported of these compounds or flavonoid-rich extracts (Mahboubi et al. 2013; Nasri et al. 2013) (Table 2 and Figure 2).

### Phenolic acids

Thirty-two (**10–42)** phenolic acid compounds with various substitutions were isolated from *S. frutescens* L. var *frutescens*, *S. canina* L., *S. takesimensis* and *S. grosheimii* (Akhmadov and Kharchenko 1969; Swiatek 1972; Swiatek and Dombrowicz 1975; Fernandez et al. 1996, 1998; Garcia et al. 1998).

**Figure 3.**
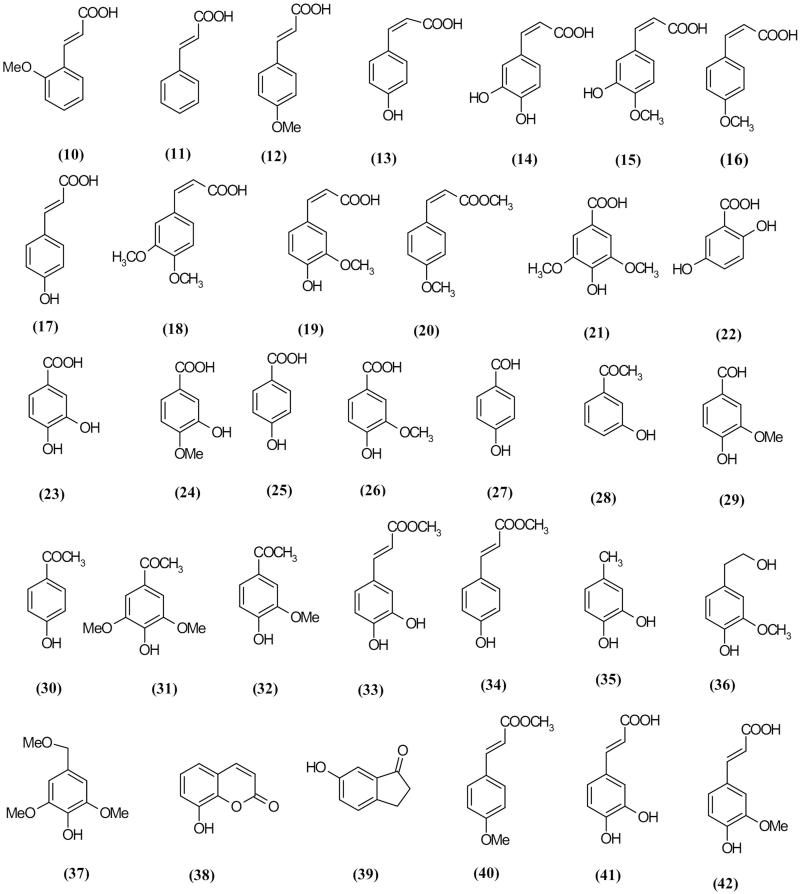
Phenolic acids compounds reported from *Scrophularia* plants.

*E*-*p*-Methoxycinnamic acid and *E*-isoferulic acid isolated from *S. buregeriana* significantly improved memory deficit, induced by scopolamine in mice (Kim et al. 2003a). *E*-*p*-Methoxycinnamic acid (Table 2 and Figure 3) also has a protective role against NMDA and glutamate-induced neurotoxicity (Kim et al. 2002b). In another experiment, *m-* and *p*-methoxycinnamic acid and ferulic acid showed hepatoprotective activities against carbon tetrachloride (CCl_4_) in animal tests (Lee et al. 2002a; Kim et al. 2011).

### Phenylethanoid glycosides

Phenylethanoid as one of the main phytochemical compounds plays specific role in biological activity of these plants. Many biological activities such as antimicrobial, anti-inflammatory, antitumor, heart function improvement and neuroprotective activities are attributed to these compounds (Zhu 1998; Koo et al. 2005; Deyama et al. 2006; Georgiev et al. 2011). Previous studies revealed that one of the main constituents of *Scrophularia* plants is phenylethanoid glycosides, and many of the therapeutic potentials can be attributed to them (Zhang and Li 2011).

**Figure 4.**
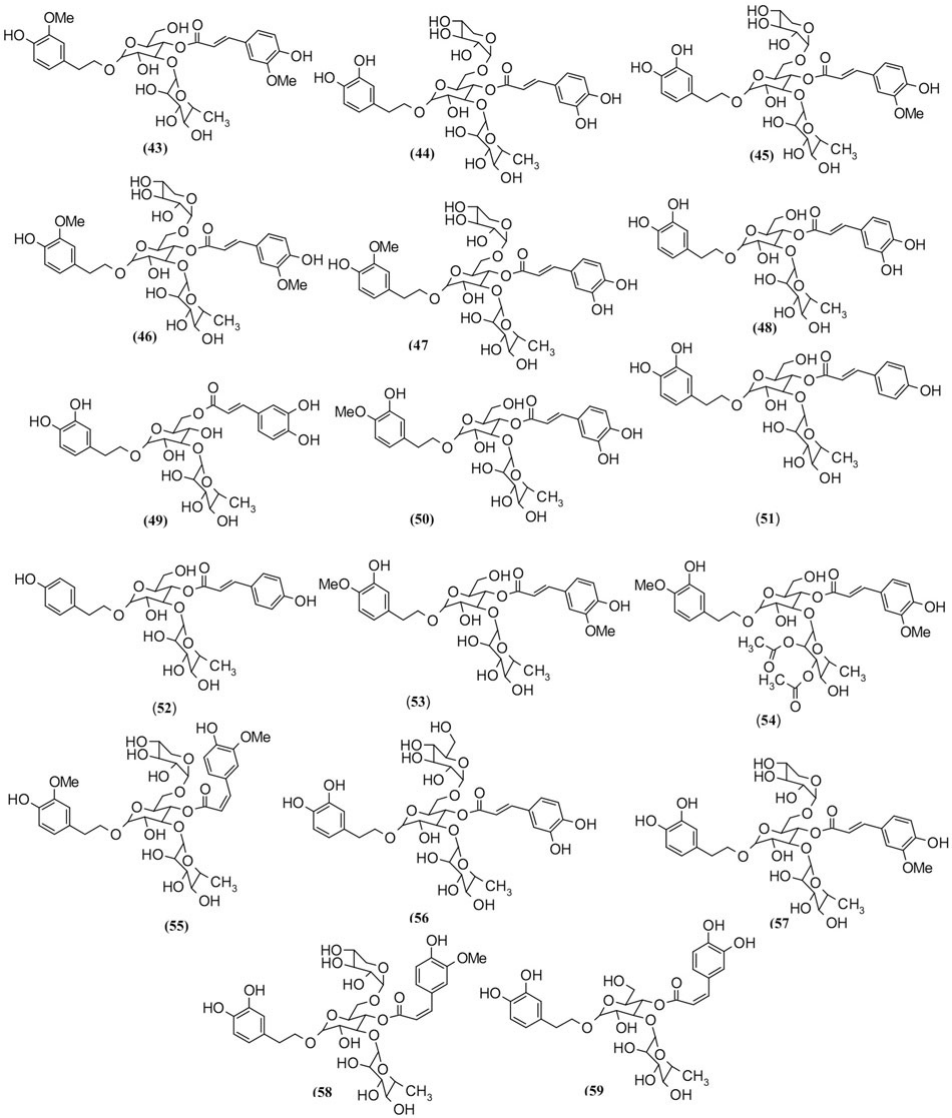
Phenylethanoid glycosides isolated from *Scrophularia* plants.

Sixteen phenylethanoid glycosides compounds (**43–59,** Figure 4) have been isolated from *Scrophularia* (Calis et al. 1988b; de Santos et al. 2000; Li et al. 2000; Lee et al. 2002a). Some of these compounds showed cytotoxicity upon investigations, for example, angoroside compounds which are isolated from *S. scopolii* Hoppe ex Pers. Among these isolated compounds, angoroside A (**39**) showed most cytotoxic activity compared with angoroside B (**40**) and angoroside C (**41**). The relationship between compound structures and their activities were elucidated.

The methoxy group on carbon (**3**) position in angoroside B and (**3**′) in angoroside C reduced cytotoxic activity compared with angoroside A (Saracoglu et al. 1997). In other research on anti-inflammatory activities of phenylpropanoids, acteoside (**43**), angoroside A (**39**) and angoroside C (**41**) have shown significant effects in TXB_2_-release assay. In addition, angoroside A (**39**), angoroside D (**42**), acteoside (**43**) and isoacteoside (**44**) significantly inhibited LPS-induced PGE_2_, NO and TNF-α (Dı´az et al. 2004). An investigation on *S. dentata* showed that phenylethanoid glycosides such as acteoside (**43**), isoacteoside (**44**), lipedosidesA-I (**51**), osmanthuside B (**52**), martynoside (**53**) and diacetylmartynoside (**54**) were isolated from this species. Phenylethanoid glycosides isolated from *Scrophularia* genus are listed in Table 2.

### Glycoside esters

**Figure 5.**
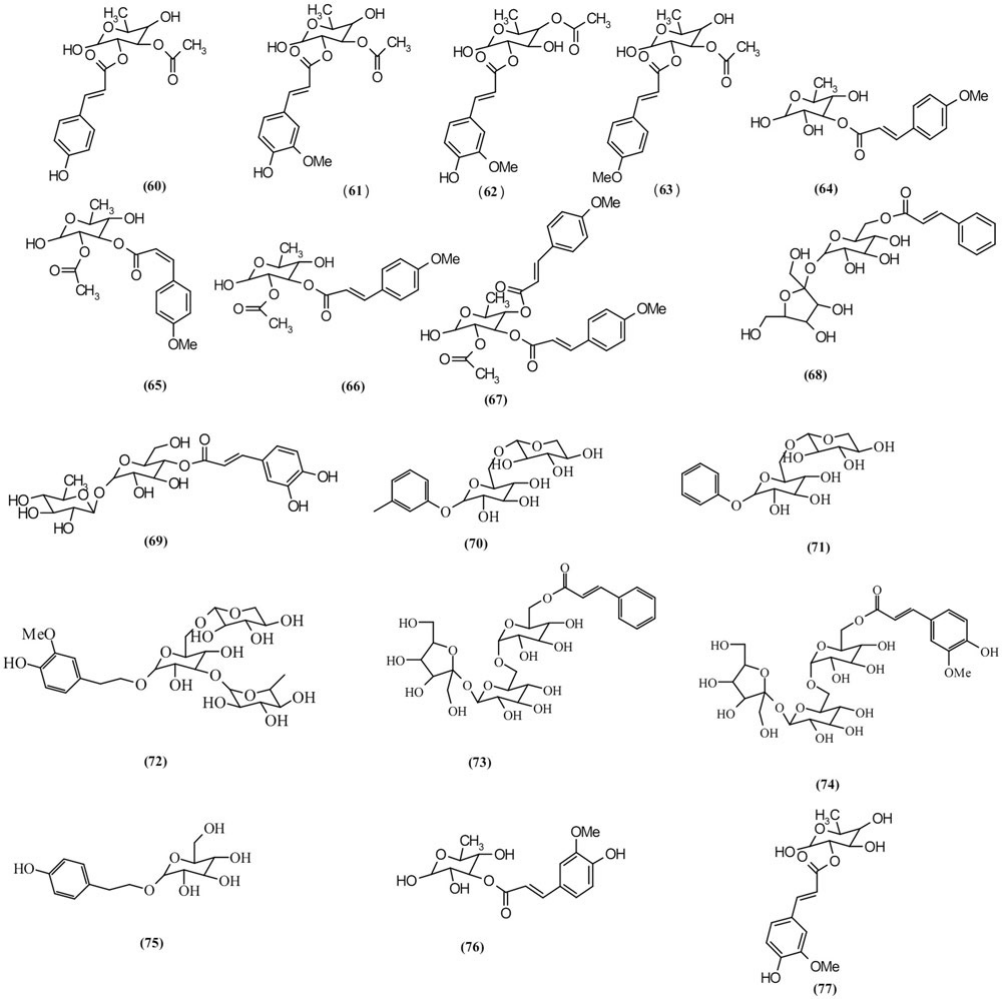
Chemical structures of *Scrophularia* glycoside esters.

Several glycoside esters (**60**–**77**, Figure 5) with various substitutions have been isolated from *S. ningpoensis* and *S. buregeriana* (Chen et al. 2007) phenylpropanoid esters of rhamnose, buergerisides A_1_, B_1_, B_2_ and C_1_ isolated from *S. buregeriana,* exhibited significant neuroprotective effects against glutamate-induced neurotoxicity (Kim and Kim 2000). Another isolated glycoside ester, ningposide D (**63**) isolated from *S. ningpoensis*, demonstrated a mild cytotoxic effect on human cancer cell line K662 on investigation (Nguyen et al. 2005). Isolated glycoside esters from various *Scrophularia* plants are listed in Table 2.

### C_9_ iridoid

**Figure 6.**
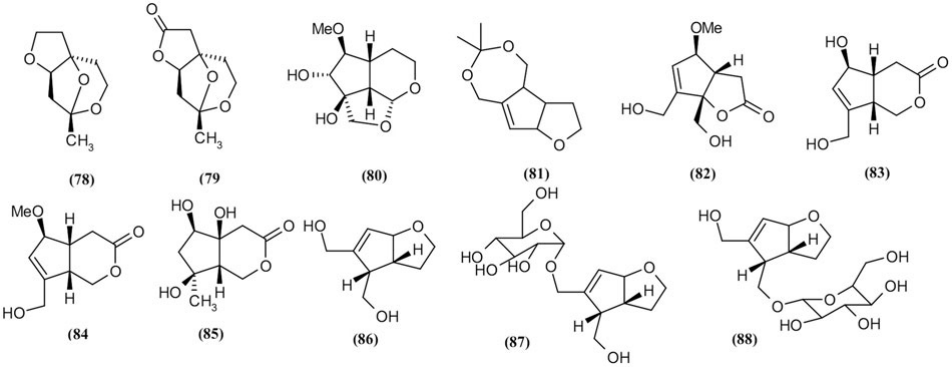
Chemical structures of *Scrophularia* C_9_ iridioides.

Several C_9_ iridoids (**78**–**88)** have been isolated from *S. buregeriana* and *S. ningpoensis*. These compounds are in glycosides and non-glycosides forms (Lin et al. 2000, 2006; Niu et al. 2009). C_9_ iridoids isolated from these plants are listed in Table 2 and Figure 6.

### Iridoid glycosides

Using different chromatography methods such as reverse phase column chromatography (RP-HPLC), size exclusion chromatography and thin layer chromatography yielded 72 iridoid glycosides from various species of *Scrophularia* (Table 2 and Figure 7) (Sticher et al. 1980; Calis et al. 1988b; Kajimoto et al. 1989; Berdini et al. 1991; Qian et al. 1991; Pachaly et al. 1994; Maksudov et al. 1996; Bermejo et al. 2002; Niu et al. 2009; Chebaki et al. 2011). Many of these compounds demonstrated various pharmacological activities such as hepatoprotective and anti-inflammatory activities (Table 4). Among the chemical compounds isolated from *S. koelzii* Pennell. such as harpagoside (**113**), koelzioside (**132**) and scropolioside A (**134**), scropolioside A demonstrated maximum hepatoprotective activity against thioacetamide-induced hepatotoxicity in animal model (Garg et al. 1994). Research on *S. deserti* led to the isolation of scropolioside D_2_ (**133**) and harpagoside B (**99**), which have significant antidiabetic and anti-inflammatory activities (Ahmed et al. 2003).

Figure 7.Chemical structures of isolated *Scrophularia* iridoid glycosides.

**Figure:**
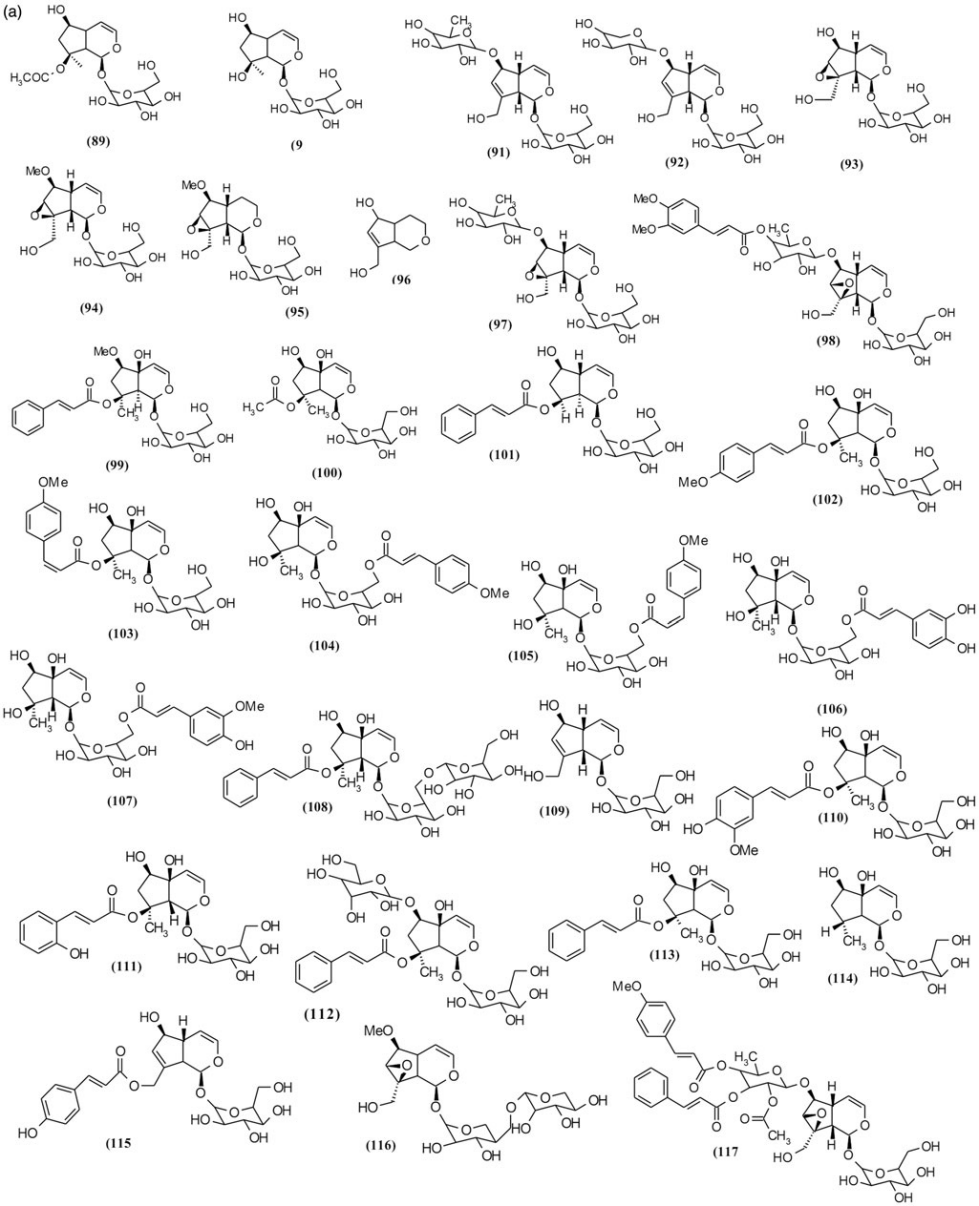

**Figure:**
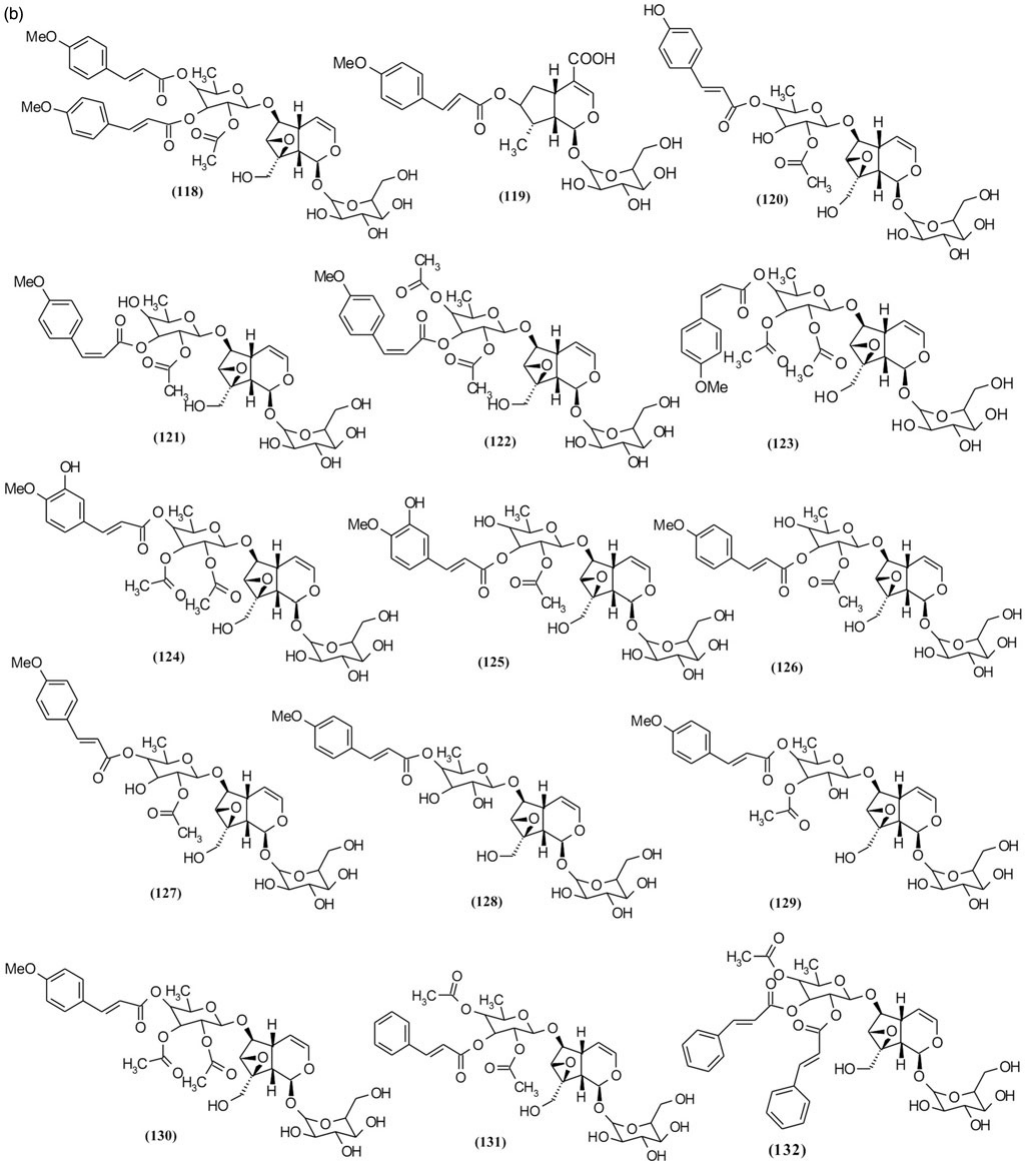

**Figure:**
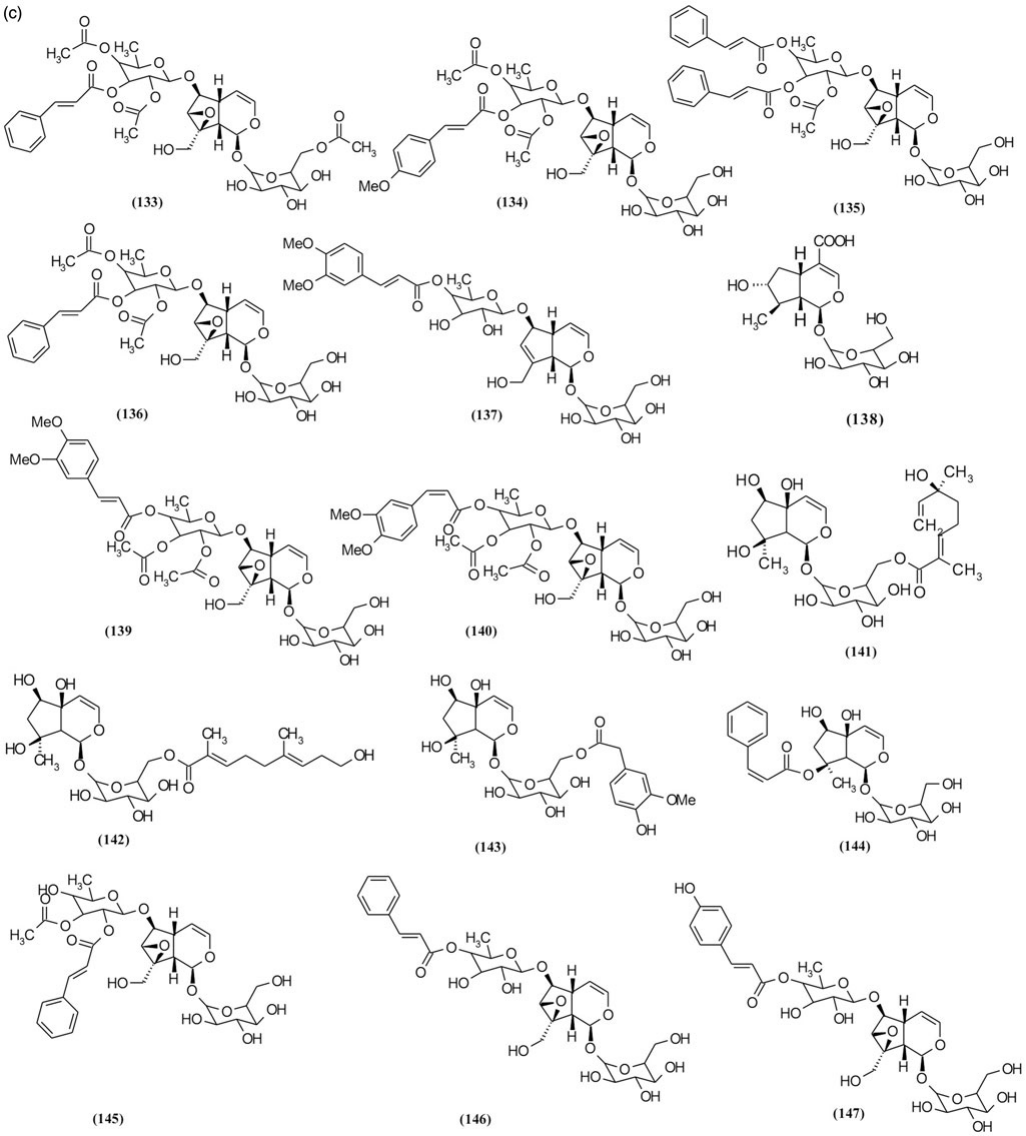

**Figure:**
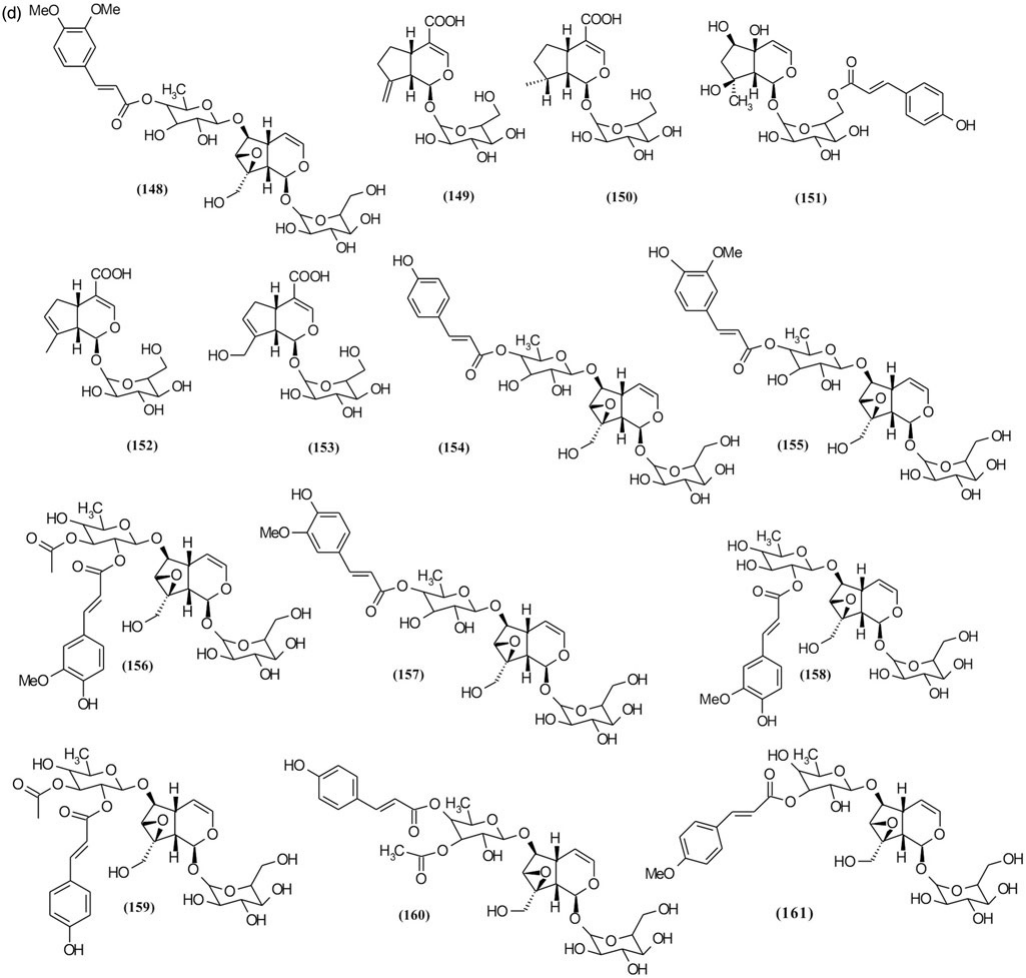

Among the various bioactivities observed of these compounds, anti-inflammatory effect is the most investigated. Zhu et al. (2015), in working on anti-inflammatory activity of isolated iridoid glycosides from *S. dentata* Royle ex Benth. and comparison between their potentials, reported their anti-inflammatory activities against LPS-induced NF-κB activity, cytokines mRNA expression, IL-1β secretion and cyclooxygenase-2 activity depending on whether the 6-*O*-substituted cinnamyl moiety was linked to C″ 2-OH, C″ 3-OH or C″ 4-OH, and on the number of moieties linked, which is closely related to the enhancement of anti-inflammatory activity (Pieroni et al. 2004). Structural diversity of iridoid glycosides in this genus can be categorized into three classes including (**a**) moieties which exist on cyclopentane ring, (**b**) moieties which exist on different position of glucose attached in [*c*] pyran ring and (**c**) moieties which exist on different position of rhamnose that are attached in C6 cyclopentane ring. Among these structural classes, diversity of iridoid glycosides with moieties in rhamnose attached in C6 cyclopentane ring position is more than other classes. Subsequently, structures with moieties are placed in different positions of cyclopentane ring, and finally structures with moieties in different positions of glucose are attached in [*c*] pyran ring. Table 2 shows various isolated *Scrophularia* iridoid glycosides.

### Alkaloids

**Figure 8.**
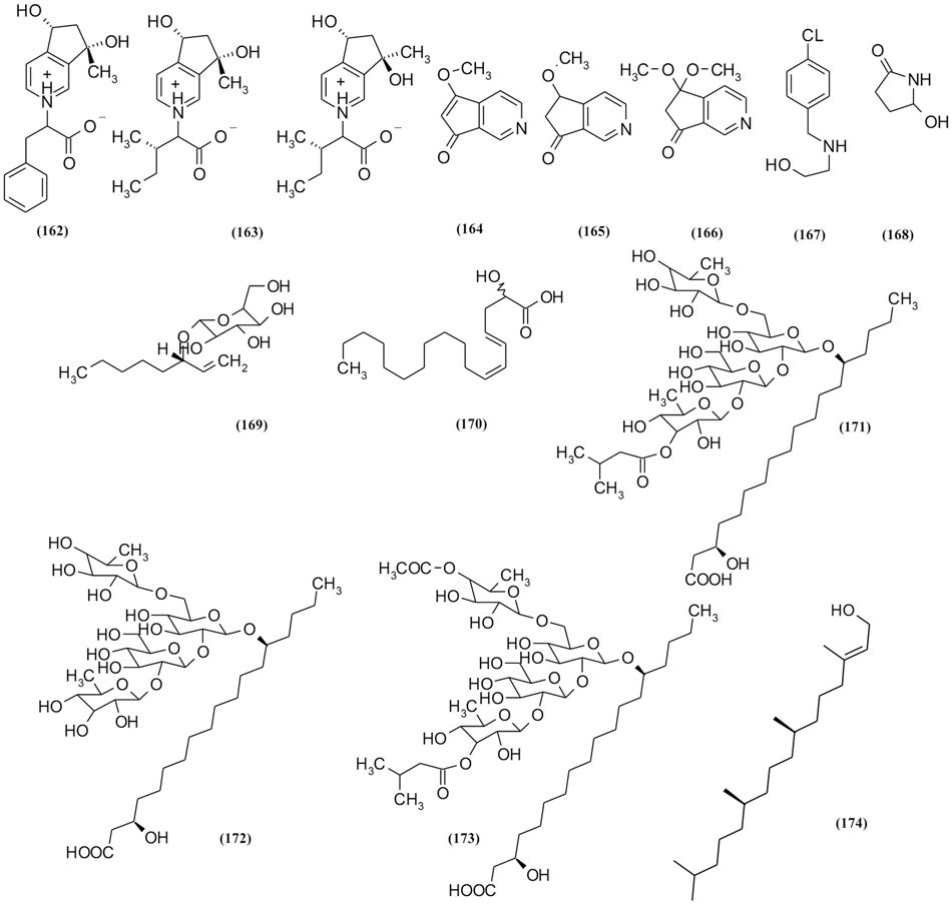
Alkaloids, resin glycosides and fatty acids derivatives of *Scrophularia* plants.

Several pyridine alkaloids are isolated from *Scrophularia* (Table 2 and Figure 8), three novel zwitterionic alkaloids-ningpoensine A **(162)** and ningpoensines B/C **(163)** (pair of epimers) were isolated from the root of *S. ningpoensis* (Zhang et al. 2015a). Ningpoensines B/C can promote wound closure in human embryonic keratinocytes in researches (Maksudov et al. 1996). In another research, three new monoterpene pyridine alkaloids, scrophularianines A–C **(164–166)** with cyclopenta [c] pyridine skeleton, were reported from *S. ningpoensis.* Other unusual new halogenated alkaloids, [2-(4-chlorobenzyl amino) ethanol] **(167)** with cytotoxic effects, were also isolated from *S. oxysepala* Boiss. (Orangi et al. 2016). Another cyclic alkaloid is 5-hydroxypyrrolidin-2-one **(168)** isolated from Korean species, *S. takesimensis* (Kim et al. 2012a).

### Resin glycosides and fatty acids derivatives

Six resin glycosides and fatty acids derivatives were isolated from *Scrophularia* (Figure 8) (Stavri et al. 2006; Çalis et al. 2007). Among these compounds, crypthophilic acids A–C **(171–173)** isolated from *S. cryptophila* Boiss. were examined for antiprotozoal and antimycobacterial activities. Crypthophilic acids A and C showed activity against *Trypanosoma brucei rhodesiense* and *Leishmania donovani* (Kajimoto et al. 1989). In another research on traditional remedy, where *S. deserti* was used as an antipyretic in Middle East countries, two unsaturated fatty acid compounds including 3(ζ)-hydroxy-octadeca-4(*E*), 6(*Z*)-dienoic acid (**170**) and 3R-1-octan-3-yl-3-*O*-β-d-glucopyranoside (**169**) were isolated. Among these compounds, 6(*Z*)-dienoic acid showed antibacterial activity against both *Staphylococcus aureus* and mycobacteria (Table 2; Ahmed et al. 2003).

### Triterpenoid glycosides and sterols

Oleanane-type triterpenoid glycoside is a major triterpenoid in *Scrophularia* species (Çalis et al. 1993b; Bhandari et al. 1996, 1997). Verbascosaponin A (**188**) as an oleanane-type triterpenoid was isolated from *S. auriculata* ssp. *pseudoauriculata* (Sennen) O. de Bolòs & J. Vigo which showed an excellent anti-inflammatory activity in the acute 12-*O*-tetradecanoylphorbol 13-acetate (TPA) model (Giner et al. 2000). In addition, three saikosaponin homologs, scrophulasaponins II–IV were isolated from *S. kakudensis* Franch. (Figure 9) (Yamamoto et al. 1993). Other isolated triterpenoid glycoside and their origin species are listed in Table 2.

Figure 9.Chemical structures of triterpenoid glycosides and sterols of *Scrophularia* species.

**Figure:**
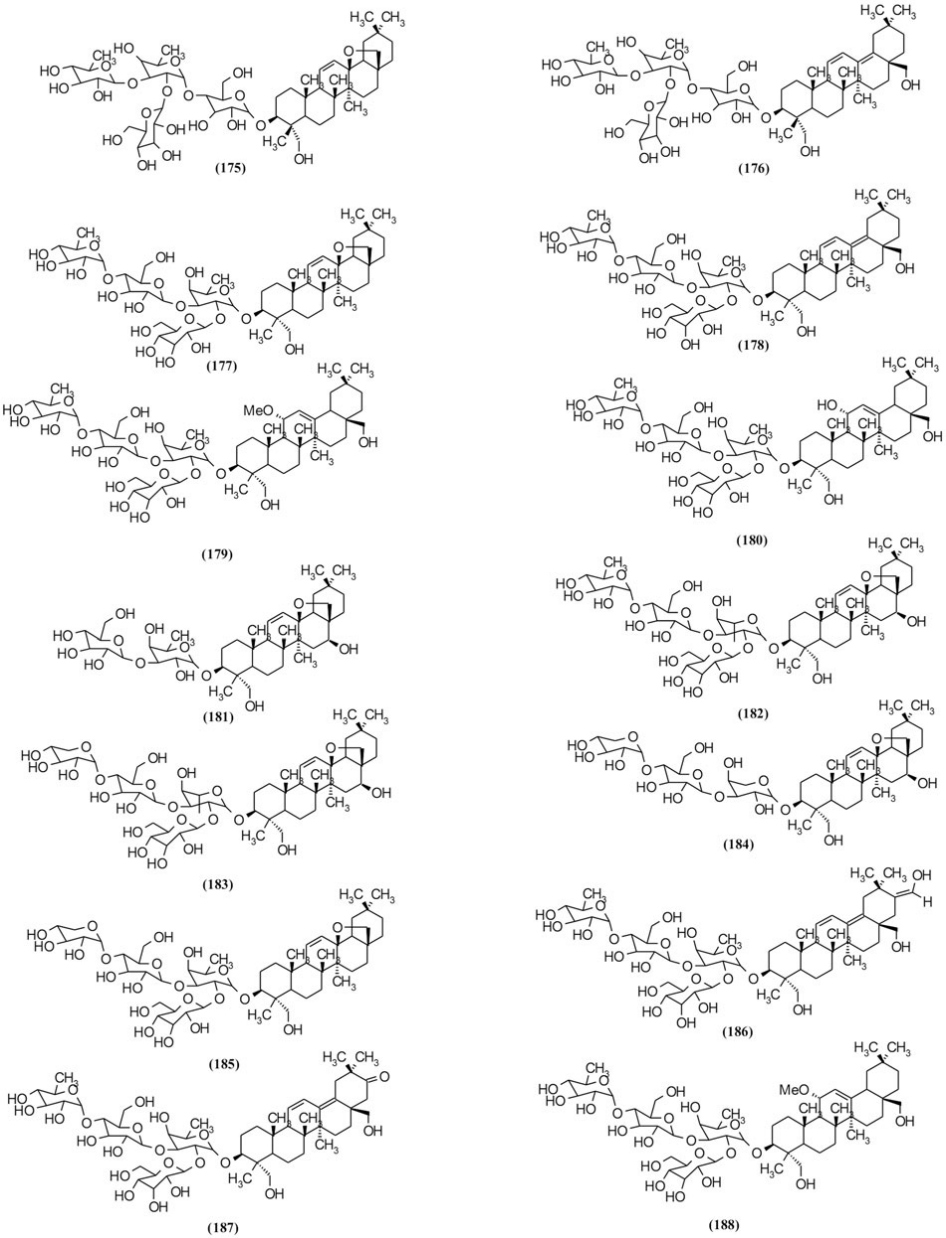

**Figure:**
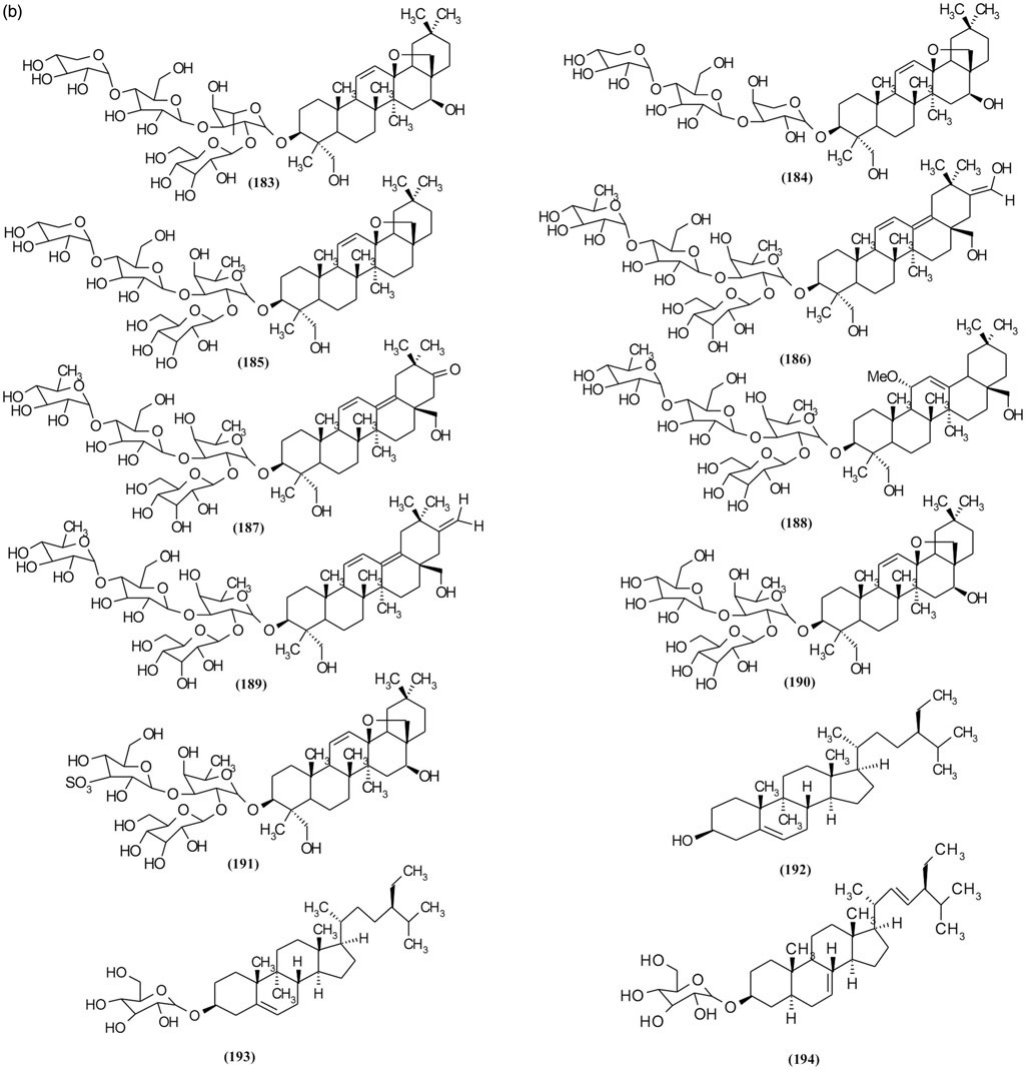

### Diterpenoids

**Figure 10.**
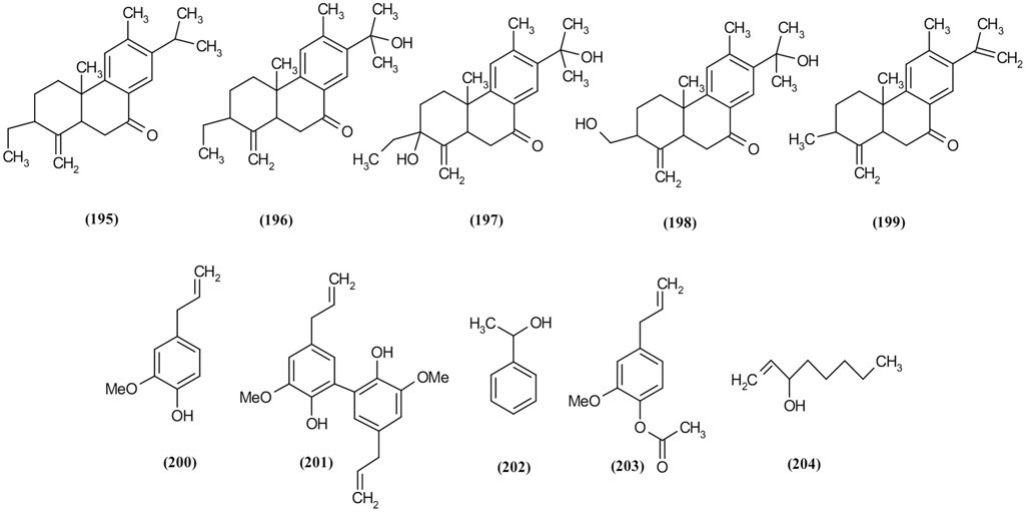
Diterpenoids and some of the essential oil major compositions of *Scrophularia* species.

Five new 19(4→3)-abeo-abietane diterpenoids, scrodentoids A–E (**195–199**) were isolated from *S. dentata,* which is a famous traditional remedy for the treatment of smallpox, measles, high-heat plague and poisoning (Zhang et al. 2015a). These compounds are isolated from low-polar extract of *S. dentata* by column chromatography and reversed-phase HPLC techniques. The anti-inflammatory, immunosuppressive, antifertility, anticystogenesis and anticancer activities of 19(4→3)-abeo-abietane diterpenoids have been previously reported (Zhang et al. 2015b). Scrodentoids A–E were investigated for immunosuppressive effect and cytotoxic effects, especially against B16 and MCF-7 cells line. According to this investigation, scrodentoids A (**195**) and D (**198**) showed the most potential in this biological test (Table 2 and Figure 10).

### Essential oils

The essential oils of a few *Scrophularia* species have been investigated until now. The essential oil of *S. oxysepala*, an endemic plant of western and central regions of Iran, was characterized by the presence of high percent of eugenol (**200**), dehydroeugenol (**201**) and methyl benzyl alcohol (**202**) as phenolic compounds. In addition, a high amount of eugenol (**200**) and eugenol acetate (**203**) have been reported from the essential oil of *S. amplexicaulis* Benth, another endemic plant of Iran, which showed antimicrobial activity against *S. aureus* (Pasdaran et al. 2012, 2013). According to research on *S. oxysepala*, *S. amplexicaulis*, *S. striata* and *S. frigida* Boiss, it was indicated that probably, 1-octen-3-ol (**204**) is a chemical compound marker in *Scrophularia* species (Table 3 and Figure 10) (Miyazawa and Okuno 2003; Amiri et al. 2011).

## Biological activity

### Anti-inflammatory

**Figure 11.**
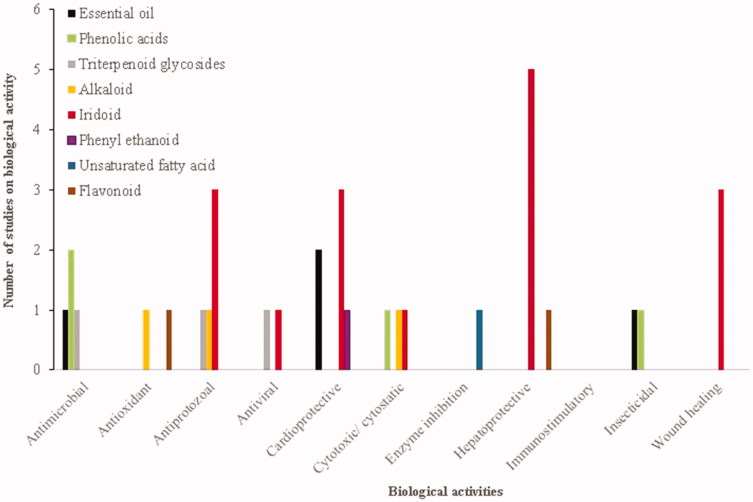
Studies on biological actives of *Scrophularia* spp. phytochemicals.

**Figure 12.**
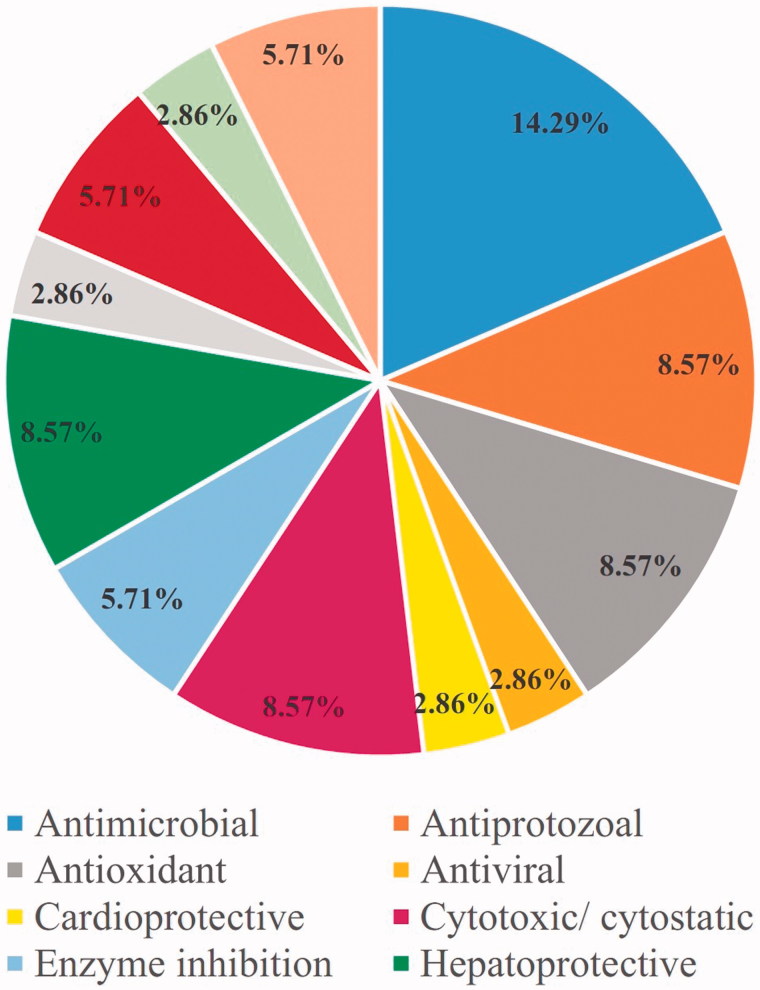
The ratio of biological activities reported for *Scrophularia* spp.

*Scrophularia denata* “Ye-Xin-Ba” a traditional Chinese herbal medicine is native to Tabatian region. The iridoids isolated from this plant showed anti-inflammatory effects in NF-κB-mediated reporter gene luciferase assay. Scropolioside B (**135**) and scropolioside D (**131**) had significant inhibitory effect against nuclear factor kappa-light-chain-enhancer of activated B cells (NF-κB) activation with an IC_50_ value of 43.7 and 1.02 μM, respectively (Zhang et al. 2014). Zhu et al. (2015) investigated the anti-inflammatory potential of various scropoliosides isolated from *S. denata* against LPS-induced NF-κB activity, cytokines mRNA expression, interleukin 1β (IL-1β) secretion and cyclooxygenase-2 activity. Scropoliosides B (**135**), F (**147**) and G (**157**) and 6-*O*-methylcatapol (**94**) significantly reduced IL-1β maturation, and secretion in the cultured medium of the THP-1 cells. Other scropoliosides A (**134**), B (**135**) and D (**131**) also inhibited IL-1β mRNA expression. Scrodentosides A and B inhibited cyclooxygenase 2 (COX-2) activity (Zhu et al. 2015). In working on *S. auriculata* ssp*. pseudoauriculata,* compounds such as verbascosaponin A (**188**) and verbascosaponin (**177**) were isolated, verbascosaponin inhibited the carrageenan paw oedema and ear oedema induced by 12-*O*-tetradecanoylphorbol 13-acetate (TPA test). Results showed that verbascosaponin A (**188**) and verbascosaponin (**177**) with an ID_50_ value of 0.32 and 0.18 µmol/ear, respectively, in comparison with indomethacin 0.35 µmol/ear have an excellent anti-inflammatory effects (Giner et al. 2000). The ethanol–water extracts of aerial parts of *S. auriculata* L. and roots of *S. buergeriana* display significant inhibition against oxazolone-induced contact-delayed hypersensitivity mouse ear oedema (DTH) and release of histamine, tumour necrosis factor-α (TNF-α), IL-4 in inflammation model, respectively (Giner et al. 2000; Kim et al. 2012b). During the investigation of *S. deserti* anti-inflammatory potential, five iridoid glycosides, including scropolioside D_2_ (**133**), harpagoside B (**99**), scropolioside D (**131**), koelzioside (**132**) and 8-*O*-acetylharpagide (**100**) were isolated and characterized (Zhu et al. 2015). Scropolioside D (**131**) and harpagoside B (**99**) isolated from *S. deserti* possess significant anti-inflammatory activity in carrageenan paw oedema (Ahmed et al. 2003). Fernandez et al. (1996, 1998) reported the anti-inflammatory activity of different extracts from *S. frutescens* L. In further screening for finding active compounds, several phenolic acids were remarkably active in the TPA test, among these isolated phenolic acid compounds, ferulic (**19**), gentisic (**22**), protocatechuic (**23**) and syringic (**21**) acids significantly inhibited oedema (protocatechuic with 71.59% inhibition; syringic with 74.43% inhibition and ferulic with 71.02% inhibition) (Fernandez et al. 1998). The roots of *S. ningpoensis* “Xuan Shen” as Chinese traditional medicine which is used against swelling, laryngitis and neuritis, consist of several iridoids and phenylethanoids, hydrophilic extract of this plant showed significant inhibitory effect (ED_50_ 20 mg/kg) on this animal model (Qian et al. 1991). *Scrophularia striata,* an Irano-Turanian region endemic plant, showed that in several anti-inflammatory models, ethyl acetate extract of *S. striata* inhibits IL-1β, TNF-α and prostaglandin E2 (PGE2) secretion in mouse peritoneal macrophages induced by lipopolysaccharide (LPS) (Figures 11 and 12; Azadmehr et al. 2013).

### Antimicrobial and antiprotozoal

Essential oil of Iranian endemic plant, *S. amplexicaulis,* showed antibacterial activity against *S. aureus* in the well diffusion method. The essential oil of this plant is characterized by a high content of eugenol (53.8%) and eugenol acetate (24.5%), and the antibacterial activity of these compounds has been identified previously (Didry et al. 1994; Pasdaran, et al. 2012). In another research on methanolic extract and fractions of *S. amplexicaulis*, 80% and 60% (IC_50_ 0.827, 0.431 mg/mL) methanol in water of solid-phase extraction (SFE) showed significant activity in haeme biocrystallization assay for potential antimalarial property (Pasdaran et al. 2016). Tasdemir et al. (2005, 2008) investigated the antiprotozoal and antimycobacterial activities of the chemical compounds of *S. cryptophila*, tryptophan and buddlejasaponin III (**184**) which showed growth-inhibitory effect against *Trypanosoma brucei* (IC_50_ 4.1 and 9.7 mg/mL). Harpagide (**114**) and crypthophilic acid C (**173**) showed the best leishmanicidal activity (IC_50_ 2.0 and 5.8 mg/mL) in comparison with other isolated compounds. In antimalarial activity against *Plasmodium falciparum,* crypthophilic acid C (**173**), tryptophan and buddlejasaponin III (**184**) showed antimalarial activity with IC_50_ values of 4.2, 16.6 and 22.4 mg/mL, respectively (Tasdemir et al. 2008). Investigation on the ethanol extract of *S. deserti* showed that plant have antibacterial potential against *Brucellla melitensis*, in other studies related to this plant, three isolated compounds including 3(ζ)-hydroxy-octadeca-4(*E*), 6(*Z*)-dienoic acid (**170**), ajugoside (**89**) and scropolioside B (**135**) exhibited moderate antibacterial activity against multidrug and methicillin-resistant *S. aureus* (MRSA) as well as mycobacteria with minimum inhibitory concentration (MIC) values, ranging from 32 to 128 µg/mL (Stavri et al. 2006; Bahmani et al. 2013). Fernandez et al. investigated the antibacterial and active fraction of *S. frutescens* and *S. sambucifolia* L. on several micro-organisms such as *Bacillus cereus*, *Bacillus megaterium*, *Bacillus subtilis*, *S. aureus*, *Escherichia coli*, *Serratia marcescens*, *Salmonella typhimurium* and *Moraxella lacunata*. Results of this investigation indicated that the phenolic fractions of both species showed more activity against Gram-positive bacteria, specifically against *Bacillus* sp. (Fernandez et al. 1996). The 70% ethanol extracts of leaves and scrokoelziside A (**175**) which were isolated from *S. ningpoensis* “Xuan Shen” showed anti-bacterial activity against *beta-haemolytic streptococci* (Figures 11 and 12; Li et al. 2009).

### Hepatoprotective and neuroprotective

*E*-*p*-Methoxycinnamic acid (**12**) isolated from *S. buergeriana* showed anti-amnesic activity and protective effect on cultured neuronal cells against neurotoxicity induced by glutamate (Kim et al. 2003a). Future investigations for finding other active compounds of *S. buergeriana* in neuroprotection led to the isolation of 10 phenylpropanoid esters from roots of this plant, although all isolated phenylpropanoid esters exerted significant protective effects against glutamate-induced neurodegeneration, but buergeriside A1 (**67**), buergeriside B1(**66**) and (*E*)-*p*-methoxycinnamic acid (**12**) exhibited better protection (Kim and Kim 2000). In the continuous isolation of other neuroprotective compounds, 8-*O*-*E*-*p*-methoxycinnamoyl harpagide (**102**) and harpagide (**114**), 8-*O-Z-* *p*-methoxycinnamoyl harpagide (**103**), 6′-*O*-E-*p*-methoxycinnamoy lharpagide (**104**), 6′-*O-*Z-p-methoxycinnamoyl harpagide (**105**) *E*-harpagoside and *Z*-harpagoside were isolated from these plants and tested for the reduction of glutamate-induced neurotoxicity in rat. According to the result, these compounds demonstrated protective effect on cultured neurons against glutamate-induced oxidative stress (Kim and Kim 2000; Kim et al. 2002a, 2003b). Isolated phenylpropanoids from roots of *S. buergeriana* exhibit hepatoprotective effect in CCl_4_-induced toxicity (Kim et al. 2002a). Chloroformic fraction of the alcoholic extract of the aerial parts of *S. koelzii* showed hepatoprotective activity. Further investigation led to the isolation of several iridoid glycosides, and among these compounds, scropolioside A showed maximum hepatoprotective activity in thioacetamide hepatotoxicity model (Figures 11 and 12; Garg et al. 1994).

## Conclusion

Recently, the amount of research on metabolites, pharmacological activities and traditional uses of the various *Scrophularia* species has increased significantly. According to reviewed literatures, several reasons could contribute to the screening of this genus which include (1) some of the species have been used as a traditional or local therapeutic remedy especially in Asia and Europe for long time, and the effectiveness and safety of these species have been established. Therefore, such sources have generated much interest and new field for easier search of potential compounds. (2) Iridoid glycosides, phenolic acids and triterpenoid glycosides have been identified as the three main chemical compositions of *Scrophularia*. Among them, scropoliosides like iridoid structures have shown potential for anti-inflammatory, hepatoprotective and wound healing activity effects. Among the less frequently isolated compounds, resin glycosides such as crypthophilic acids have shown good properties in antiprotozoal and antibacterial assays. Therefore, chemical compounds of this genus will motivate further investigation on *Scrophularia,* and have great potential as sources of finding new therapeutic medications. (3) Only 17 of the approx. 350 species have been studied in some detail. Among the isolated metabolites from *Scrophularia* spp., only a few of them has been investigated for their biological activities. Many of the conducted researches on isolation or biological screening have been conducted on iridoids and phenylethanoids while other classes of phytochemicals such as alkaloids, diterpenoids and flavonoids have been less considered by researchers.

On one hand, most of the studies on the isolated compounds have been carried and *in vitro/in vivo* and we could not find any clinical trials on biological activities of *Scrophularia*. Thus, pharmacokinetic and metabolism of these metabolites are unclear in human body. On the other hand, the exact mechanism of the active isolated molecules is still unknown. Considering these issues, there is huge gap between the current situation and the final goal which is developing approved drug from the isolated molecules or even developing supplements from the *Scrophularia* spp. extracts. Conducting ADME (absorption, distribution, metabolism and excretion) studies on the isolated bioactive compound of the genus seems to be essential.

In most cases, quantitative analysis of bioactive compounds has not been considered which might guide researchers to find other species of *Scrophularia* with more content of bioactive compounds. Despite the presence of some *Scrophularia* species in different pharmacopeias and their application in tradition or folk medicine of different societies, lack of analytical investigations on the bioactive compounds of these species resulted in difficulties in quality control and standardizations of these herbs.

Some metabolites, such as iridoids which also demonstrated some biological activities, are common between these plants and it is possible to consider them as biomarkers for *Scrophularia* spp.

Conducting complementary studies on isolated bioactive compound from this genus, such as Quantitative structure–activity relationship (QSAR) studies on the isolated bioactive compounds as well as preparing semi-synthetic derivatives, may result in more active metabolites.

## References

[CIT0001] Ahmed B, Al-Rehaily AJ, Al-Howiriny TA, El-Sayed KA, Ahmad MS. 2003 Scropolioside-D2 and harpagoside-B: two new iridoid glycosides from *Scrophularia deserti* and their antidiabetic and antiinflammatory activity. Biol Pharm Bull. 26:462–467. 12673026 10.1248/bpb.26.462

[CIT0002] Akhmadov SGTDA, Kharchenko NS. 1969 Pharmacology of flavonoid aglicons of *Scrophularia grosheimi* . Farmakol Toksikol 32:693–694. 5381600

[CIT0003] Akhmedov S, Litvinenko V. 1969 Scrophulein—a new flavonoid from *Scrophularia grossheimii* . Chem Nat Compd. 5:47–48.

[CIT0004] Amiri H, Lari Y, Esmaeili A, Samsamnia F, Eghbali D, Viskarami G, Dosti B, Noormohamadi E. 2011 Essential oil composition and anatomical study of *Scrophularia striata* Boiss. IJMAP. 27:271–278.

[CIT0005] Azadmehr A, Afshari A, Baradaran B, Hajiaghaee R, Rezazadeh S, Monsef-Esfahani H. 2009 Suppression of nitric oxide production in activated murine peritoneal macrophages *in vitro* and *ex vivo* by *Scrophularia striata* ethanolic extract. J Ethnopharmacol. 124:166–169. 19527828 10.1016/j.jep.2009.03.042

[CIT0006] Azadmehr A, Maliji G, Hajiaghaee R, Shahnazi M, Afaghi A. 2013 Inhibition of pro-inflammatory cytokines by ethyl acetate extract of *Scrophularia striata* . Trop J Pharm Res. 11:893–897.

[CIT0007] Bahmani M, Vakili-Saatloo N, Maghsoudi R, Momtaz H, Saki K, Kazemi-Ghoshchi B. 2013 A comparative study on the effect of ethanol extract of wild *Scrophularia deserti* and streptomycin on *Brucellla melitensis* . J HerbMed Pharmacol. 2:17–20.

[CIT0008] Bahrami A, Ali V. 2010 Effects of *Scrophularia striata* ethanolic leaves extracts on *Staphylococcus aureus* . Int J Pharmacol. 6:431–434.

[CIT0009] Benito PB, Martinez MA, Sen AS, Gómez AS, Matellano LF, Contreras SS, Lanza AD. 1998 *In vivo* and *in vitro* antiinflammatory activity of saikosaponins. Life Sci. 63:1147–1156. 9763210 10.1016/s0024-3205(98)00376-2

[CIT0010] Berdini R, Bianco A, Guiso M, Marini E, Nicoletti M, Passacantilli P, Righi G. 1991 Isolation and partial synthesis of 7, 8-dehydro-6β, 10-dihydroxy-11-noriridomyrmecin, a methylcyclopentanoid monoterpene from *Scrophularia canina* . J Nat Prod. 54:1400–1403.

[CIT0011] Bermejo P, Abad MJ, Díaz AM, Fernández L, De Santos J, Sanchez S, Villaescusa L, Carrasco L, Irurzun A. 2002 Antiviral activity of seven iridoids, three saikosaponins and one phenylpropanoid glycoside extracted from *Bupleurum rigidum* and *Scrophularia scorodonia* . Planta Med. 68:106–110. 11859457 10.1055/s-2002-20238

[CIT0012] Bhandari S, Babu U, Garg H. 1997 A triterpene glycoside from *Scrophularia koelzii* . Phytochemistry. 45:1717–1719. 10.1016/0031-9422(95)00697-4 8835460

[CIT0013] Bhandari S, Roy R, Agrawal P, Garg H. 1996 Scrokoelziside A, a triterpene glycoside from *Scrophularia koelzii* . Phytochemistry. 41:879–882. 8835460 10.1016/0031-9422(95)00697-4

[CIT0014] Bhandri S, Mishra A, Roy R, Garg H. 1992 Koelzioside, an iridoid diglycoside from *Scrophularia koelzii* . Phytochemistry. 31:689–691.

[CIT0015] Calis I, Gross G-A, Sticher O. 1988a Two phenylpropanoid glycosides from *Scrophularia scopolii* . Phytochemistry. 27:1465–1468.

[CIT0016] Calis I, Gross G-A, Winkler T, Sticher O. 1988b Isolation and structure elucidation of two highly acylated iridoid diglycosides from *Scrophularia scopolii* . Planta Med. 54:168–170. 17265233 10.1055/s-2006-962382

[CIT0017] Çalis I, Sezgin Y, Dönmez AA, Rüedi P, Tasdemir D. 2007 Crypthophilic acids A, B, and C: resin glycosides from aerial parts of *Scrophularia crypthophila* L. J Nat Prod. 70:43–47. 17253848 10.1021/np060511k

[CIT0018] Çalis I, Zor M, Basaran AA, Wright AD, Sticher O. 1993a Karsoside and scropolioside D, two new iridoid glycosides from *Scrophularia ilwensis* . J Nat Prod. 56:606–609. 8496707 10.1021/np50094a022

[CIT0019] Çalis İ, Zor M, Başaran AA, Wright AD, Sticher O. 1993b Ilwensisaponins A, B, C, and D: triterpene saponins from *Scrophularia ilwensis* . Helv Chim Acta. 76:1352–1360.

[CIT0020] Chebaki R, Haba H, Long C, Marcourt L, Benkhaled M. 2011 Acylated iridoid glycosides from *Scrophularia saharae* Batt. & Trab. Biochem Syst Ecol. 39:902–905.

[CIT0021] Chen B, Liu Y, Liu HW, Wang NL, Yang BF, Yao XS. 2008 Iridoid and aromatic glycosides from *Scrophularia ningpoensis* Hemsl. and their inhibition of Ca^2+^ i increase induced by KCl. Chem Biodivers. 5:1723–1735. 18816525 10.1002/cbdv.200890161

[CIT0022] Chen B, Wang N, Huang J, Yao X. 2007 Iridoid and phenylpropanoid glycosides from *Scrophularia ningpoensis* Hemsl. Asian J Tradit Med. 2:118–123.

[CIT0023] Commission CP 2005. Pharmacopoeia of the People's Republic of China. China: Beijing People's Medical Publ House.

[CIT0024] Cuéllar M, Giner R, Recio M, Just M, Máñez S, Cerda S, Ríos J. 1998 Screening of antiinflammatory medicinal plants used in traditional medicine against skin diseases. Phytother Res. 12:18–23.

[CIT0025] de Santos J, Lanza AMD, Fernández L, Rumbero A. 2000 Isoangoroside C, a phenylpropanoid glycoside from *Scrophularia scorodonia* roots. Z Naturforsch C J Biosci. 55:333–336. 10.1515/znc-2000-5-606 10928542

[CIT0026] Deyama T, Kobayashi H, Nishibe S, Tu P. 2006 Isolation, structure elucidation and bioactivities of phenylethanoid glycosides from *Cistanche*, *Forsythia* and *Plantago* plants. Stud Nat Prod Chem. 33:645–674.

[CIT0027] Dı´az AMa, Abad MJ, Fernández L, Silván AM, De Santos J, Bermejo P. 2004 Phenylpropanoid glycosides from *Scrophularia scorodonia*: *in vitro* anti-inflammatory activity. Life Sci. 74:2515–2526. 15010262 10.1016/j.lfs.2003.10.008

[CIT0028] Didry N, Dubreuil L, Pinkas M. 1994 Activity of thymol, carvacrol, cinnamaldehyde and eugenol on oral bacteria. Pharm Acta Helv. 69:25–28. 7938073 10.1016/0031-6865(94)90027-2

[CIT0029] Dinda B, Chowdhury DR, Mohanta BC. 2009 Naturally occurring iridoids, secoiridoids and their bioactivity. An updated review, part 3. Chem Pharm Bull. 57:765–796. 19652401 10.1248/cpb.57.765

[CIT0030] Emam AM, Diaz-Lanza AM, Matellano FL, Faure R, Moussa AM. 1997 Biological activities of buddlejasaponin isolated from *Buddleja madagascariensis* and *Scrophularia scorodonia* . Pharmazie. 52:76–77. 9035239

[CIT0031] Fernandez M, Saenz M, Garcia M. 1998 Natural products: anti-inflammatory activity in rats and mice of phenolic acids isolated from *Scrophularia frutescens* . J Pharm Pharmacol. 50:1183–1186. 9821668 10.1111/j.2042-7158.1998.tb03332.x

[CIT0032] Fernandez MA, Garcia M, Saenz M. 1996 Antibacterial activity of the phenolic acids fractions of *Scrophularia frutescens* and *Scrophularia sambucifolia* . J Ethnopharmacol. 53:11–14. 8807471 10.1016/0378-8741(96)01419-5

[CIT0033] Galindez JS, Fernández Matellano L, Lanza AMD. 2001 Iridoids from *Scrophularia* genus. Z Naturforsch C J Biosci. 56:513–520. 10.1515/znc-2001-7-807 11531083

[CIT0034] Garcia M, Ahumada M, Saenz M. 1998 Cytostatic activity of some phenolic acids of *Scrophularia frutescens* L. var. *frutescens* . Z Naturforsch C. 53:1093–1095.

[CIT0035] Garg H, Bhandari S, Tripathi S, Patnaik G, Puri A, Saxena R, Saxena R. 1994 Antihepatotoxic and immunostimulant properties of iridoid glycosides of *Scrophularia koelzii* . Phytother Res. 8:224–228.

[CIT0036] Garrido G, González D, Lemus Y, Garcıa D, Lodeiro L, Quintero G, Delporte C, Núñez-Sellés AJ, Delgado R. 2004 *In vivo* and *in vitro* anti-inflammatory activity of *Mangifera indica* L. extract (VIMANG®). Pharmacol Res. 50:143–149. 15177302 10.1016/j.phrs.2003.12.003

[CIT0037] Georgiev M, Alipieva K, Orhan I, Abrashev R, Denev P, Angelova M. 2011 Antioxidant and cholinesterases inhibitory activities of *Verbascum xanthophoeniceum* Griseb. and its phenylethanoid glycosides. Food Chem. 128:100–105. 25214335 10.1016/j.foodchem.2011.02.083

[CIT0038] Germinara GS, Frontera AM, De Cristofaro A, Rotundo G. 2011 Insecticidal activity of different extracts from *Scrophularia canina* L. against *Culex pipiens molestus* Forskal (Diptera, Culicidae). J Environ Sci Health B. 46:473–479. 21726144 10.1080/03601234.2011.583858

[CIT0039] Giner R, Ma Villalba Ma L, Recio Ma C, Máñez S, Cerdá-Nicolás M, Rı´os J-L. 2000 Anti-inflammatory glycoterpenoids from *Scrophularia auriculata* . Eur J Pharmacol. 389:243–252. 10688990 10.1016/s0014-2999(99)00846-8

[CIT0040] Giner RM, Villalba ML, Recio MdC Máñez S, Gray AI, Ríos JL. 1998 A new iridoid from *Scrophularia auriculata *ssp.* pseudoauriculata* . J Nat Prod. 61:1162–1163. 9748391 10.1021/np980067o

[CIT0041] Goodyer J, Gunther RT. 1968. The Greek herbal of Dioscorides. London: Hafner Pub. Co. Illustrated by a Byzantine, A.D. 512; English ed. by John Goodyer, A.D. 1677; edited and first printed, A.D. 1933. “Facsimile of the 1934 edition.”

[CIT0042] Guarrera PM, Lucia LM. 2007 Ethnobotanical remarks on central and southern Italy. J Ethnobiol Ethnomed. 3:23. 17537240 10.1186/1746-4269-3-23 PMC1906747

[CIT0043] Hajiaghaee R, Monsef Esfahani HR, Khorramizadeh MR, Saadat F, Shahverdi AR, Attar F. 2007 Inhibitory effect of aerial parts of *Scrophularia striata* on matrix metalloproteinases expression. Phytother Res. 21:1127–1129. 17639554 10.1002/ptr.2221

[CIT0044] Jeong EJ, Lee KY, Kim SH, Sung SH, Kim YC. 2008 Cognitive-enhancing and antioxidant activities of iridoid glycosides from *Scrophularia buergeriana* in scopolamine-treated mice. Eur J Pharmacol. 588:78–84. 18462717 10.1016/j.ejphar.2008.04.015

[CIT0045] Kajimoto T, Hidaka M, Shoyama K, Nohara T. 1989 Iridoids from *Scrophularia ningpoensis* . Phytochemistry. 28:2701–2704.

[CIT0046] Kim H-Y, Park J, Lee K-H, Lee D-U, Kwak J-H, Kim YS, Lee S-M. 2011 Ferulic acid protects against carbon tetrachloride-induced liver injury in mice. Toxicology. 282:104–111. 21291945 10.1016/j.tox.2011.01.017

[CIT0047] Kim H, Ahn M, Lee S. 2012a Isolation and identification of phytochemical constituents from *Scrophularia takesimensis* . J Med Plant Res. 6:3923–3930.

[CIT0048] Kim J-K, Kim YH, Lee HH, Lim SS, Park KW. 2012b Effect of *Scrophularia buergeriana* extract on the degranulation of mast cells and ear swelling induced by dinitrofluorobenzene in mice. Inflammation. 35:183–191. 21318391 10.1007/s10753-011-9304-x

[CIT0049] Kim SR, Kang SY, Lee KY, Kim SH, Markelonis GJ, Oh TH, Kim YC. 2003a Anti-amnestic activity of *E-p*-methoxycinnamic acid from *Scrophularia buergeriana* . Brain Res Cogn Brain Res. 17:454–461. 12880915 10.1016/s0926-6410(03)00161-7

[CIT0050] Kim SR, Kim YC. 2000 Neuroprotective phenylpropanoid esters of rhamnose isolated from roots of *Scrophularia buergeriana* . Phytochemistry. 54:503–509. 10939354 10.1016/s0031-9422(00)00110-2

[CIT0051] Kim SR, Koo KA, Sung SH, Ma CJ, Yoon JS, Kim YC. 2003b Iridoids from *Scrophularia buergeriana* attenuate glutamate-induced neurotoxicity in rat cortical cultures. J Neurosci Res. 74:948–955. 14648601 10.1002/jnr.10828

[CIT0052] Kim SR, Lee KY, Koo KA, Sung SH, Lee N-G, Kim J, Kim YC. 2002a Four new neuroprotective iridoid glycosides from *Scrophularia buergeriana* roots. J Nat Prod. 65:1696–1699. 12444706 10.1021/np0202172

[CIT0053] Kim SR, Sung SH, Jang YP, Markelonis GJ, Oh TH, Kim YC. 2002b E-p-methoxycinnamic acid protects cultured neuronal cells against neurotoxicity induced by glutamate. Br J Pharmacol. 135:1281–1291. 11877337 10.1038/sj.bjp.0704576 PMC1573240

[CIT0054] Koo KA, Sung SH, Park JH, Kim SH, Lee KY, Kim YC. 2005 *In vitro* neuroprotective activities of phenylethanoid glycosides from *Callicarpa dichotoma* . Planta Med. 71:778. 16142646 10.1055/s-2005-871213

[CIT0055] Korkina L, Mikhal’chik E, Suprun M, Pastore S, Dal Toso R. 2007 Molecular mechanisms underlying wound healing and anti-inflammatory properties of naturally occurring biotechnologically produced phenylpropanoid glycosides. Cell Mol Biol (Noisy-Le-Grand). 53:84–91. 17543237

[CIT0056] Lee E, Kim S, Kim J, Kim Y. 2002 Hepatoprotective phenylpropanoids from *Scrophularia buergeriana* roots against CCl_4_-induced toxicity: action mechanism and structure–activity relationship. Planta Med. 68:407–411. 12058315 10.1055/s-2002-32081

[CIT0057] Lewis W, Edinburgh RCoPo, Edinburgh RHa. 1748 The Pharmacopoeia of the Royal College of the Physicians at Edinburgh, Materia Medica. 4th ed. London: John Nourse.

[CIT0058] Li J, Huang X, Du X, Sun W, Zhang Y. 2009 Study of chemical composition and antimicrobial activity of leaves and roots of *Scrophularia ningpoensis* . Nat Prod Res. 23:775–780. 19418360 10.1080/14786410802696247

[CIT0059] Li Y-M, Jiang S-H, Gao W-Y, Zhu D-Y. 2000 Phenylpropanoid glycosides from *Scrophularia ningpoensis* . Phytochemistry. 54:923–925. 11014290 10.1016/s0031-9422(00)00171-0

[CIT0060] Lin S-J, Jiang S-H, Li Y-m, Zeng J-F, Zhu D-Y. 2000 Two novel iridoids from *Scrophularia buergeriana* . Tetrahedron Lett. 41:1069–1071.

[CIT0061] Lin SJ, Tan CH, Jiang SH, Li YM, Zhu DY. 2006 C9 Iridoids from *Scrophularia buergeriana* . Hca. 89:2789–2793.

[CIT0062] Mahboubi M, Kazempour N, Nazar ARB. 2013 Total phenolic, total flavonoids, antioxidant and antimicrobial activities of *Scrophularia striata* Boiss extracts. Jundishapur J Nat Pharm Prod. 8:15–19. 24624181 PMC3941883

[CIT0063] Maksudov M, Saatov Z, Abdullaev N. 1996 Iridoids of *Scrophularia leucoclada* plants. Chem Nat Compd. 32: 212.

[CIT0064] Marty AT. 1999 The complete German commission E monographs: therapeutic guide to herbal medicines. JAMA. 281:1852–1853.

[CIT0065] Ministry of Health tFR 2012 French Pharmacopoeia, Scrofulosa nodosa for homoeopathic preparations. Paris: L'Adrapharm.

[CIT0066] Miyase T, Mimatsu A. 1999 Acylated iridoid and phenylethanoid glycosides from the aerial parts of *Scrophularia nodosa* . J Nat Prod. 62:1079–1084. 10479307 10.1021/np9805746

[CIT0067] Miyazawa M, Okuno Y. 2003 Volatile components from the roots of *Scrophularia ningpoensis* Hemsl. Flavour Fragr J. 18:398–400.

[CIT0068] Monsef-Esfahani HR, Hajiaghaee R, Shahverdi AR, Khorramizadeh MR, Amini M. 2010 Flavonoids, cinnamic acid and phenyl propanoid from aerial parts of *Scrophularia striata* . Pharm Biol. 48:333–336. 20645822 10.3109/13880200903133829

[CIT0069] Nasri S, Cheraghi J, Soltanbaygi S. 2013 Antinociceptive and anti-inflammatory effect of alcoholic extract of root and stem of *Scrophularia striata* Boiss. in male mice. IJMAP. 29:Pe74–Pe83.

[CIT0070] Nguyen A-T, Fontaine J, Malonne H, Claeys M, Luhmer M, Duez P. 2005 A sugar ester and an iridoid glycoside from *Scrophularia ningpoensis* . Phytochemistry. 66:1186–1191. 15924924 10.1016/j.phytochem.2005.03.023

[CIT0071] Niu Z-R, Wang R-F, Shang M-Y, Cai S-Q. 2009 A new iridoid glycoside from *Scrophularia ningpoensis* . Nat Prod Res. 23:1181–1188. 19731136 10.1080/14786410802386344

[CIT0072] Orangi M, Baradaran B, Pasdaran A, Kazemi T, Hosseini B, Mohammadnejad L. 2013 Cytotoxic effect of methanolic fractions of *Scrophularia oxysepala* on MCF-7 human breast cancer cell line. Front Immunol Conference Abstract: 15th International Congress of Immunology (ICI). doi: 103389/conf fimmu.591.

[CIT0073] Orangi M, Pasdaran A, Shanehbandi D, Kazemi T, Yousefi B, Hosseini B-A, Baradaran B. 2016 Cytotoxic and apoptotic activities of methanolic subfractions of *Scrophularia oxysepala* against human breast cancer bell line. Evid Based Complement Alternat Med. 2016:1–10. 10.1155/2016/8540640 PMC4789403 27034697

[CIT0074] Pachaly P, Barion J, Sin K. 1994 Isolierung und strukturaufklärung neuer iridoidglycoside aus *Crophularia koraiensis* . Pharmazie. 49:150–155.

[CIT0075] Pasdaran A, Delazar A, Ayatollahi SA, Nahar L, Sarker SD. 2016 Phytochemical and bioactivity evaluation of *Scrophularia amplexicaulis* Benth. Rec Nat Prod. 10:519–526.

[CIT0076] Pasdaran A, Delazar A, Ayatollahi SA, Pasdaran A. 2017 Chemical composition and biological activities of methanolic extract of *Scrophularia oxysepala* Boiss. Iran J Pharm Res. 16:338. 28496487 PMC5423259

[CIT0077] Pasdaran A, Delazar A, Nazemiyeh H, Nahar L, Sarker SD. 2012 Chemical composition, and antibacterial (against *Staphylococcus aureus*) and free-radical-scavenging activities of the essential oils of *Scrophularia amplexicaulis* Benth. Rec Nat Prod. 6:350–355.

[CIT0078] Pasdaran A, Nahar L, Asnaashari S, Sarker SD, Delazar A. 2013 GC-MS analysis, free-radical-scavenging and insecticidal activities of essential oil of *Scrophularia oxysepala* Boiss. Pharm Sci. 19:1.

[CIT0079] Pharmacopoeia SJ. 2006 The Japanese Pharmacopoeia. 15 ed. Tokyo: Yakuji Nippo.

[CIT0080] Pieroni A, Quave CL, Santoro RF. 2004 Folk pharmaceutical knowledge in the territory of the Dolomiti Lucane, inland southern Italy. J Ethnopharmacol. 95:373–384. 15507363 10.1016/j.jep.2004.08.012

[CIT0081] Pinkas MTF, Peng W, Torck M. 1994 Use, chemistry and pharmacology of ten Chinese medicinal plants. Fitoterapia. 65:343–354.

[CIT0082] Qian J, Hunkler D, Rimpler H. 1992 Iridoid-related aglycone and its glycosides from *Scrophularia ningpoensis* . Phytochemistry. 31:905–911.

[CIT0083] Qian J, Hunkler D, Safayhi H, Rimpler H. 1991 New iridoid-related constituents and the anti-inflammatory activity of *Scrophularia ningpoensis* . Planta Med. 57:A56.

[CIT0084] Ramunno A, Serrilli Am Piccioni F, Serafini M, Ballero M. 2006 Taxonomical markers in two endemic plants of Sardinia: *Verbascum conocarpum* and *Scrophularia trifoliata* . Nat Prod Res. 20:511–516. 16644551 10.1080/14786410600677423

[CIT0085] Saracoglu I, Calis I, Inoue M, Ogihara Y. 1997 Selective cytotoxic and cytostatic activity of some phenylpropanoid glycosides. Fitoterapia. 68:434–438.

[CIT0086] Stavri M, Mathew K, Gibbons S. 2006 Antimicrobial constituents of *Scrophularia deserti* . Phytochemistry. 67:1530–1533. 16797620 10.1016/j.phytochem.2006.05.011

[CIT0087] Stevenson PC, Simmonds MS, Sampson J, Houghton PJ, Grice P. 2002 Wound healing activity of acylated iridoid glycosides from *Scrophularia nodosa* . Phytother Res. 16:33–35. 11807962 10.1002/ptr.798

[CIT0088] Sticher O, Meier B, Lehmann D, Świątek L. 1980 Scrophulariosid, ein neues iridoidglucosid aus *Scrophularia lateriflora* . Planta Med. 38:246–254.

[CIT0089] Swiatek L. 1972 Phenolic acids of underground parts of *Scrophularia nodosa* L. Pol J Pharmacol Pharm. 25:461–464. 4777350

[CIT0090] Swiatek L, Dombrowicz E. 1975 Pharmacobotanical investigations on some species of the Scrophulariaceae family. Part V. Chemical constituents in *Lathraea squamaria* L. Pol J Pharmacol Pharm. 28:105–109. 1264854

[CIT0091] Tasdemir D, Brun R, Franzblau SG, Sezgin Y, Çalıs I. 2008 Evaluation of antiprotozoal and antimycobacterial activities of the resin glycosides and the other metabolites of *Scrophularia cryptophila* . Phytomedicine. 15:209–215. 17761408 10.1016/j.phymed.2007.07.032

[CIT0092] Tasdemir D, Güner ND, Perozzo R, Brun R, Dönmez AA, Çalıs I, Rüedi P. 2005 Anti-protozoal and plasmodial FabI enzyme inhibiting metabolites of *Scrophularia lepidota* roots. Phytochemistry. 66:355–362. 15680992 10.1016/j.phytochem.2004.11.013

[CIT0093] Valiyari S, Baradaran B, Delazar A, Pasdaran A, Zare F. 2012 Dichloromethane and methanol extracts of *Scrophularia oxysepala* induces apoptosis in MCF-7 human breast cancer cells. Adv Pharm Bull. 2:223. 24312797 10.5681/apb.2012.034 PMC3845997

[CIT0094] Venditti A, Frezza C, Riccardelli M, Foddai S, Nicoletti M, Serafini M, Bianco A. 2015 Secondary metabolites from *Scrophularia canina* L. Nat Prod Res. 1–5: 1665–1669. 10.1080/14786419.2015.1122598 26675659

[CIT0095] Viola S. 1966 Plante Medicinale e Velenose de la Flora ltaliana. Milan: Maestri. p. 179.

[CIT0096] Wang S, Li Y, Devinsky O, Schachter S, Pacia S. 2005 Traditional Chinese medicine. In: Complementary and Alternative Therapies for Epilepsy. New York, NY: Demos Medical Pub. p. 177–182.

[CIT0097] Willoughby MJ, Mills S, Committee BHMAS. 1996 British Herbal Pharmacopoeia. 4th ed England: British Herbal Medicine Association.

[CIT0098] Yamamoto A, Miyase T, Ueno A, Maeda T. 1993 Scrophulasaponins II-IV, new saikosaponin homologs from *Scrophularia kakudensis* FRANCH. Chem Pharm Bull. 41:1780–1783. 8281574 10.1248/cpb.41.1780

[CIT0099] Yan X, Xie G. 2011. Encyclopedia of traditional Chinese medicines: molecular structures, pharmacological activities, natural sources and applications; Volume 1 AC.

[CIT0100] Zhang J, Ip FC, Tong EP, Chan KW, Li L-C, Ng YP, Ip NY. 2015a Ningpoensines A-C: unusual zwitterionic alkaloids from *Scrophularia ningpoensis* . Tetrahedron Lett. 56:5453–5456.

[CIT0101] Zhang L, Guo F, Wang S, Li Y. 2012 A new triterpenoid tetrasaccharide from the root of *Scrophularia ningpoensis* . Yao Xue Xue Bao. 47:1358–1362. 23289149

[CIT0102] Zhang L, Li Y. 2011 Advances in studies on chemical constituents in plants of *Scrophularia* L. and their pharmacological effects in recent ten years. Zhong Cao Yao. 42:2360–2368.

[CIT0103] Zhang L, Zhang D, Jia Q, Wang R, Dorje G, Zhao Z, Guo F, Yang Y, Li Y. 2015b 19(4→3)-abeo-abietane diterpenoids from Scrophularia dentata Royle ex Benth. Fitoterapia. 106:72–77. 26291646 10.1016/j.fitote.2015.08.005

[CIT0104] Zhang L, Zhu T, Qian F, Xu J, Dorje G, Zhao Z, Guo F, Li Y. 2014 Iridoid glycosides isolated from *Scrophularia dentata* Royle ex Benth. and their anti-inflammatory activity. Fitoterapia. 98:84–90. 25016952 10.1016/j.fitote.2014.07.005

[CIT0105] Zhu L-J, Hou Y-L, Shen X-Y, Pan X-D, Zhang X, Yao X-S. 2013 Monoterpene pyridine alkaloids and phenolics from *Scrophularia ningpoensis* and their cardioprotective effect. Fitoterapia. 88:44–49. 23602903 10.1016/j.fitote.2013.04.005

[CIT0106] Zhu T, Zhang L, Ling S, Qian F, Li Y, Xu J-W. 2015 Anti-Inflammatory activity comparison among Scropoliosides–Catalpol derivatives with 6-*O*-substituted cinnamyl moieties. Molecules. 20:19823–19836. 26540037 10.3390/molecules201119659 PMC6331811

[CIT0107] Zhu Y-P. 1998 Chinese materia medica: chemistry, pharmacology and applications. Amsterdam: Amsterdam Harwood Academic Publishers.

